# Histone Methyltransferase SETD1B Maintains Cancer Stem Cell Niche by Regulating the Crosstalk between CD24 and Surface Adhesion Molecules in Hepatocellular Carcinoma

**DOI:** 10.7150/ijbs.112943

**Published:** 2025-07-24

**Authors:** Yan Gao, Wei Zhou, Yuehong Gao, Shunxi Wang, Zhiling Xu, Xiao Xiang, Li Yang

**Affiliations:** 1 National Innovation and Attracting Talents “111” base, Key Laboratory of Biorheological Science and Technology, Ministry of Education, College of Bioengineering, Chongqing University, Chongqing, 400044, China.; 2 Department of Radiation Oncology, Chongqing University Cancer Hospital, Chongqing, China.; 3 College of Biology and Environmental Sciences, Jishou University, Jishou, China.

**Keywords:** HCC, Liver cancer stem cells, SETD1B, Stemness, Triptolide

## Abstract

**Purpose:** Hepatocellular carcinoma (HCC) is a highly aggressive malignancy with a dismal prognosis that is largely attributed to the capacity of liver cancer stem cells (LCSCs) to self-renew in response to conventional therapies. Therefore, it is crucial to develop new therapeutic strategies that target LCSCs to improve the clinical outcomes of patients with HCC.

**Experimental Design:** We surveyed and analyzed publicly available single-cell TCGA (the cancer genome atlas), single-cell (scRNA-seq) and spatial RNA-sequencing databases from HCC patient specimens for genes uniquely expressed in LCSCs. We generated and characterized LCSCs from patient-derived HCC cell lines and used them as tools to uncover the previously unknown molecular mechanisms associated with the stemness of LCSCs. We selectively screened a bank of natural compounds to identify drugs that can specifically target LCSCs for HCC treatment and documented their effects both *in vitro* and *in vivo*.

**Results:** TCGA analyses showed that SETD1B expression was aberrantly elevated in HCC, correlating with poor prognosis and a distinct molecular signature of stemness. We demonstrated that SETD1B, driven by MAZ, enhances stem characteristics by promoting anchorage-independence, cellular adhesion, tumor sphere formation, and growth via the surface glycoprotein CD24. We identified triptolide (Trip), which serves as a potent suppressor of LCSC stemness by targeting SETD1B for degradation, thereby dramatically attenuating HCC progression *in vitro* and *in vivo*.

**Conclusions:** These findings establish the MAZ/SETD1B/CD24 signaling cascade as a critical regulatory mechanism of LCSC stemness and highlight Trip as a potential therapeutic agent for HCC.

## Introduction

Hepatocellular carcinoma (HCC) is the most common primary malignancy of the liver, accounting for approximately 75-85% of all liver cancer cases globally, and its incidence rate is expected to continue to rise in the next 30 years 1. HCC is amongst the top six most prevalent types of malignancies and the third leading cause of cancer-related deaths worldwide 2, 3. While HCC typically arises in the context of chronic liver disease and cirrhosis, with major etiological risk factors including chronic hepatitis B and C virus infections, recent years of scientific research on the underlying genetic and molecular pathways have led to the development of targeted therapies 4, 5. Although the introduction of these newer treatment modalities, such as inclusion of lenvatinib, a kinase inhibitor, as a mono- or in combinatorial therapy, has moderately improved the survival of the patients in recent years, due to unclear reasons, the majority of patients still experience tumor recurrence and metastasis 6, 7.

Liver cancer stem cells (LCSCs) represent a subpopulation of HCC cell that possess self-renewal and differentiation capabilities, contributing to tumor initiation, progression, metastasis, and resistance to conventional therapies 8, 9. These cells exhibit distinct phenotypic markers, including EpCAM, CD90, CD133, and CD44, which are associated with stem-like properties and often linked to poor prognosis and aggressive clinical outcomes 10, 11. Increasing evidence suggests that the resurgence of tumor cells is due to the incomplete eradication of cancer stem cells; thus, targeted depletion of these cell populations has been a major strategy for the treatment of HCC 12, 13. However, the precise regulatory pathways and signaling mechanisms governing LCSC maintenance remain unclear, necessitating further investigation to develop effective LCSC-specific therapeutic strategies.

The maintenance of LCSC stemness is driven by a complex interplay of molecular mechanisms that sustain self-renewal, differentiation potential, and tumorigenic capacity, in which epigenetic regulation has emerged as a major determinant 14, 15. In cancer, DNA methylation patterns are often dysregulated, with hypermethylation of tumor suppressor genes and hypomethylation of oncogenic pathways contributing to unchecked proliferation, invasion, and metastasis 16. This dysregulated methylation landscape not only facilitates tumor development but also plays a central role in maintaining cancer stem cell (CSC) populations 17. Aberrant DNA methylation patterns in the promoter regions of tumor suppressor genes, for instance, can lead to their silencing, enhancing LCSC survival and proliferation. Additionally, histone modifications, including acetylation and methylation, modulate chromatin structure and accessibility, impacting transcriptional programs that support stemness and differentiation pathways in LCSCs 18.

Here we report a novel epigenetic mechanism that governs LCSC properties and drug resistance. SETD1B (SET Domain Containing 1B) is a histone methyltransferase that is frequently overexpressed or mutated, leading to aberrant H3K4me3 deposition and dysregulation of pathways that promote oncogenesis 19, 20. However, its role in HCC has not been explored. Intrigued by its high expression in malignant tissues and close association with poor survival, we generated LCSC surrogates to study SETD1B function. Using a combination of scRNA-seq analysis, spatial transcriptomics, and *in vivo* animal models, we found that SETD1B maintained LCSC stemness and induced carcinogenesis by dramatically enhancing anchorage-independent clonal expansion via the CD24 receptor signaling cascade. Promoter analyses revealed that this high SETD1B level was controlled by MYC-associated zinc finger protein (MAZ), a multifunctional protein involved in transcriptional regulation. More importantly, we demonstrated that SETD1B can be targeted for degradation by triptolide, a naturally occurring diterpenoid epoxide found in the gold vine, which leads to remarkable control of tumor growth, even when used as a monotherapy in our preclinical model of HCC.

## Results

### SETD1B is associated with the stem-like properties of hepatocellular carcinoma

**Figure 1:**
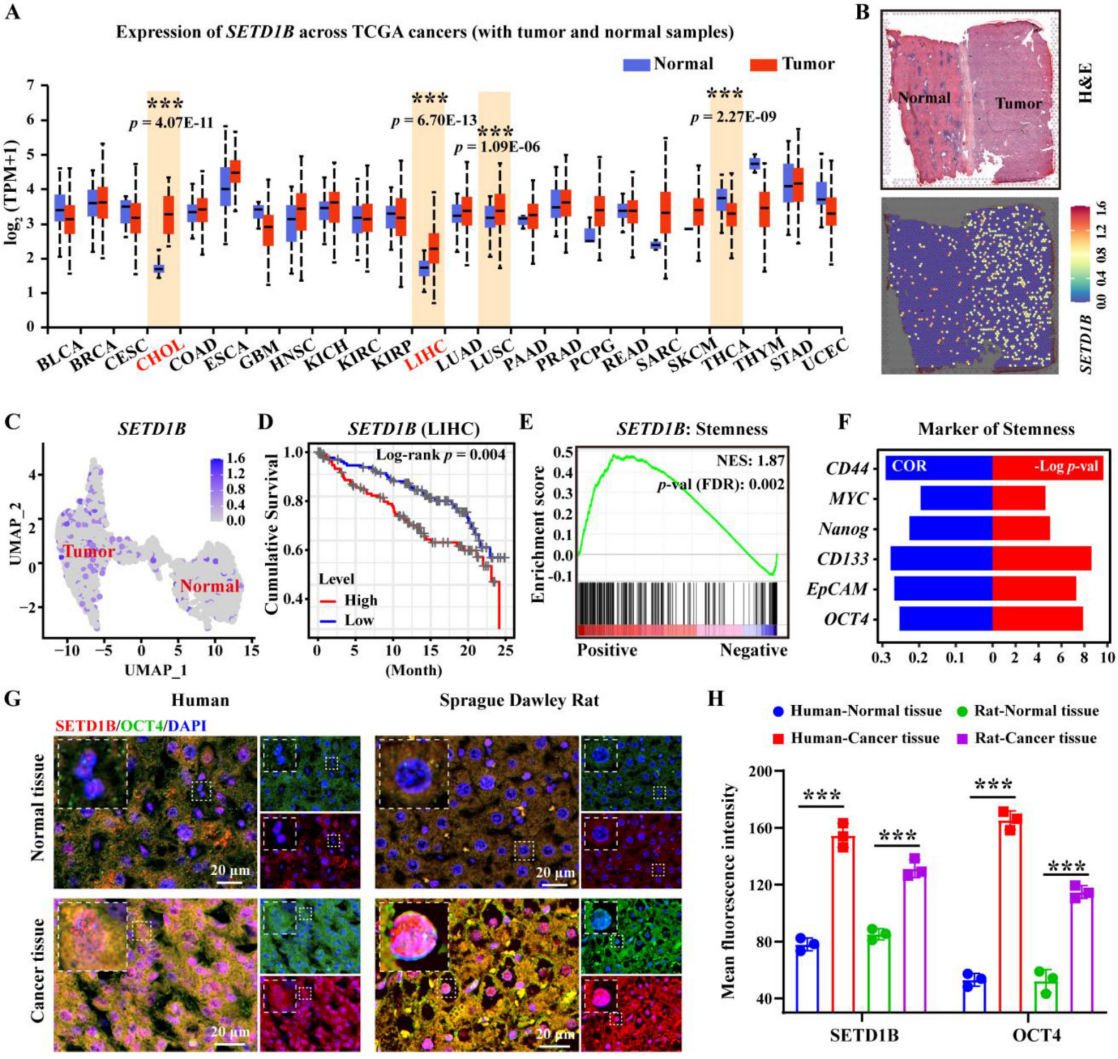
**SETD1B is associated with the stem-like properties of hepatocellular carcinoma (HCC). (A)** The pan-cancer-view of *SETD1B*. **(B)** Spatial transcriptomics (HRA000437) analysis of *SETD1B* expression in liver tumor-adjacent tissue and HCC tissue. **(C)** The UMAP of spatial transcriptomics (HRA000437) analysis showing the expression of *SETD1B* in in liver tumor-adjacent tissue and HCC tissue. **(D)** Overall survival prognosis of *SETD1B* in liver cancer. **(E)** Enrichment of stemness signaling pathways associated with *SETD1B*. **(F)** Correlation and *p*-value ranking of stemness-related markers *CD44*, *MYC*,* Nanog*, *CD133*, *EpCAM*, and *OCT4* with *SETD1B*. **(G)** Immunofluorescence (IF) staining of SETD1B and OCT4 in normal and tumor specimens. **(H)** Quantification of SETD1B and OCT4 fluorescence in each normal and tumor specimens. ** *p* < 0.01, *** *p* < 0.001.

To investigate the role of SETD1B in liver cancer, we assessed its expression characteristics using TCGA database. Our unbiased pan-cancer expression analysis revealed that *SETD1B* levels were significantly altered in a number of cancer tissues compared with their respective normal counterparts (**Figure 1A**; **Figure S1A**). Surprisingly, *SETD1B* expression was most significantly elevated in hepatocellular carcinoma (HCC, referred to as LIHC in TCGA) and cholangiocarcinoma (CHOL) (**Figure 1A**; **Figure S1A**-**C**). This was further corroborated by the spatial transcriptomics (HRA000437) of patient tumor specimens, which showed a clear difference in *SETD1B* expression in HCC as opposed to its adjacent normal tissues (**Figure 1B** and** C**; **Figure S1D** and** E**). Notably, even the early stages of LIHC and CHOL exhibited dramatic up-regulation of *SETD1B* expression, which remained high across all stages (**Figure S1F** and** G**). Importantly, high *SETD1B* expression levels were associated with a poor prognosis in patients with HCC (**Figure 1D**). These findings suggested that SETD1B plays a crucial role in the development and progression of HCC. As SETD1B is involved in regulating the differentiation of hematopoietic stem cells, we wondered whether it contributed to the maintenance of LCSCs (liver cancer stem cells) 21, 22. As we expected, gene set enrichment analysis (GSEA) revealed a statistically significant (*p* < 0.05) positive correlation between *SETD1B* and pathways governing cell stemness and the expression of various classical stem cell markers (**Figure 1E** and** F**; **Figure S1H**). To confirm this correlation, we obtained HCC and matched normal tissue specimens from both human patients and Sprague-Dawley (SD) rat orthotopic liver cancer models. Using OCT4 as a stemness indicator, our immunofluorescence (IF) analyses demonstrated simultaneous significant up-regulation of OCT4 and SETD1B in cancerous tissues compared to normal tissues in specimens of both human and rat origin (**Figure 1G** and **H**). Together, these results indicated that SETD1B is closely related to the stemness of LCSCs, warranting further investigations.

### SETD1B is highly expressed in liver cancer stem cells

**Figure 2:**
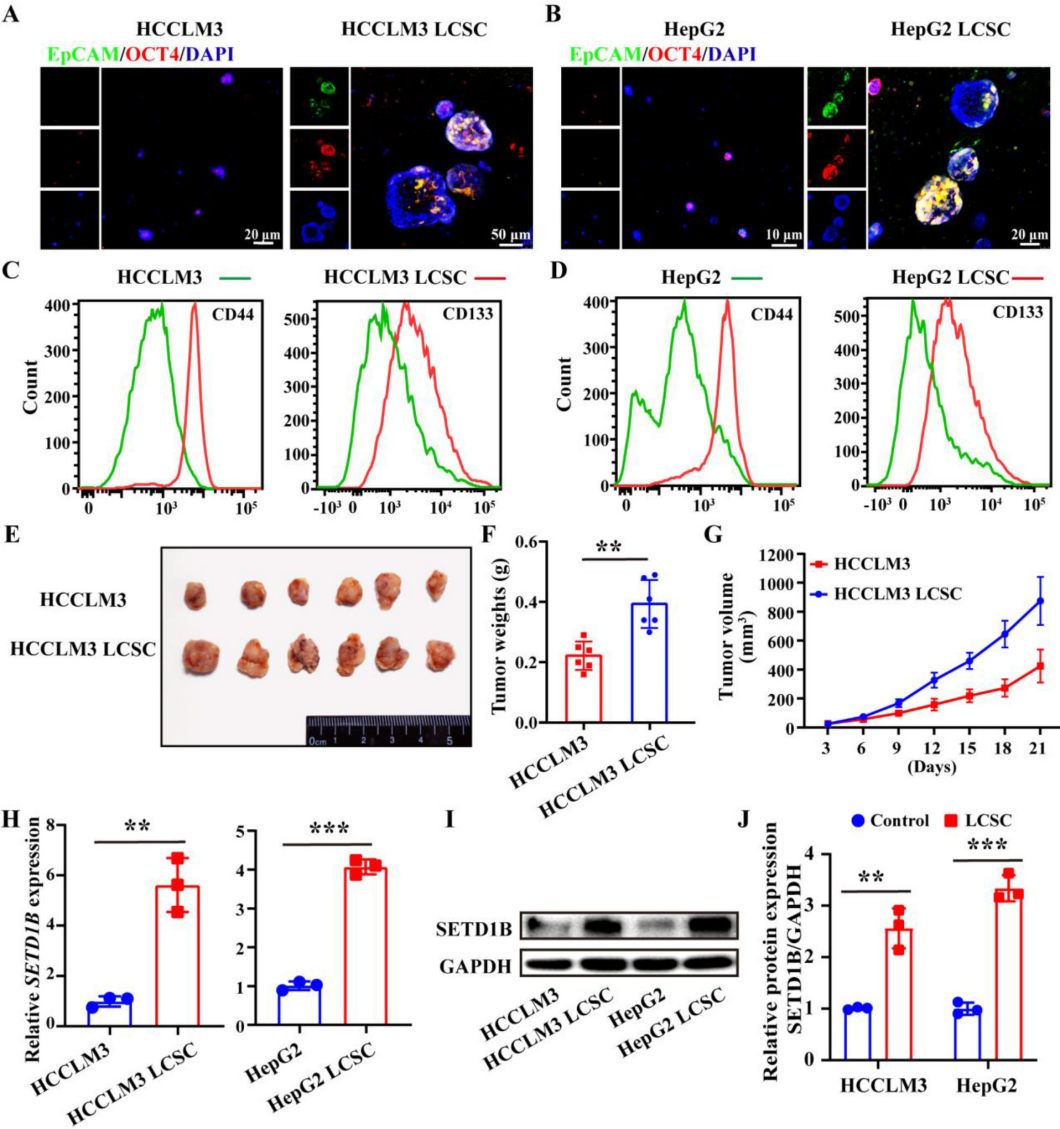
**SETD1B is highly expressed in liver cancer stem cells (LCSCs). (A) (B)** IF staining of EpCAM and OCT4 in HCCLM3/HepG2 cells and LCSCs. **(C) (D)** Flow cytometry (FC) detection of stemness markers (CD4 and CD133) in HCCLM3/HepG2 cells and LCSCs. **(E)** Tumor formation capabilities of HCCLM3 cells and HCCLM3 LCSCs in nude mice. **(F)** Statistical analysis of tumor weight. **(G)** Statistical analysis of tumor volumes. **(H)** Gene expression of *SETD1B* in HCCLM3/HepG2 cells and LCSCs. **(I)** Protein expression of SETD1B in HCCLM3/HepG2 cells and LCSCs. **(J)** Quantitative image analysis of SETD1B expression. ** p < 0.01, *** *p* < 0.001.

To reveal the functional role of SETD1B in LCSCs, we dedifferentiated the HCC cell lines, HCCLM3 and HepG2, by continuous culture in non-adhesion plates in a stem cell growth medium. After a 21-day self-reprogramming period, both treated HCCLM3 and HepG2 cells, now denoted as HCCLM3 and HepG2 LCSCs, started expressing epithelial cell adhesion molecule (EpCAM) and OCT4, which are characteristic markers of LCSC compared to their wild-type counterparts (**Figure 2A** and** B**). To further complement our IF assessment, we evaluated several other classical markers of stemness such as CD44, CD133, Nanog, EpCAM, SOX2 and OCT4 by flow cytometry (**Figure 2C** and **D**), RT-qPCR (**Figure S2A** and **B**), and Western blotting (**Figure S2C**-**F**) in both treated and wild-type HCCLM3 and HepG2 cells. Furthermore, we demonstrated the enhanced tumorigenic capacity of the induced HCCLM3 LCSCs compared to its parent control *in vivo* by showing accelerated tumor growth and significantly larger tumors at the endpoint in nude mice (**Figure 2E**-**G**; **Figure S2G**-**I**). Collectively, these results indicate the successful induction of LCSC in HCCLM3 and HepG2 cell lines. Next, we investigated whether SETD1B levels were altered in both the cell lines after stem cell induction. Using RT-qPCR and Western blotting, we found that SETD1B mRNA and protein levels were both dramatically increased in the HCCLM3 and HepG2 LCSCs groups compared with their respective parental controls (**Figure 2H**-**J**). These findings align with previous pathological analyses of patient HCC specimens and clearly demonstrate high SED1B expression in LCSCs.

### SETD1B was involved in regulating the stemness of liver cancer stem cells

**Figure 3:**
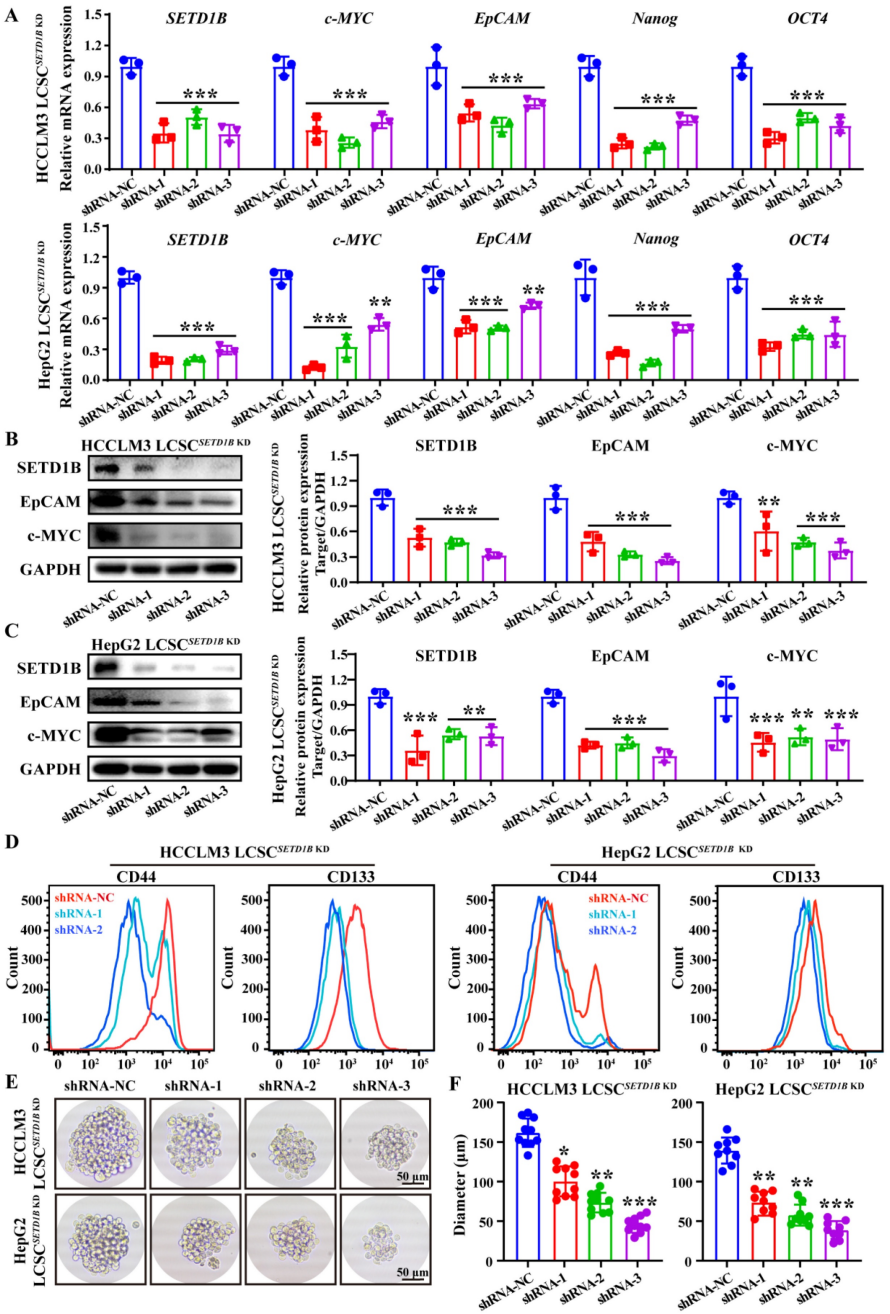
** SETD1B maintains stem cell characteristics in liver cancer cells. (A)** Expression of *SETD1B* and stemness markers genes (*c*-*MYC*, *EpCAM*, *Nanog* and* OCT4*) in HCCLM3 and HepG2 LCSCs after *SETD1B* KD. **(B) (C)** Expression and quantification of SETD1B, EpCAM and c-MYC proteins in HCCLM3 and HepG2 LCSCs after *SETD1B* KD. **(D)** FC detection of stemness markers (CD44, CD133) in HCCLM3 and HepG2 LCSCs after *SETD1B* KD. **(E)** Assessment of spheroid formation ability in HCCLM3 and HepG2 LCSCs after *SETD1B* KD. **(F)** Quantification of the diameters in HCCLM3 and HepG2 LCSCs after *SETD1B* KD. * p < 0.05, ** p < 0.01, *** p < 0.001

However, does SETD1B regulate the stemness of LCSCs? To answer this question, we assessed the changes in LCSC characteristics after *SETD1B* knockdown (KD) using RNA interference. Remarkably, after short hairpin RNA (shRNA)-mediated reduction of *SETD1B*, we observed significant down-regulation of all markers of stemness tested, including c-MYC, EpCAM, Nanog, and OCT4, in both HCCLM3 and HepG2 LCSCs compared to those in the non-targeting controls (NC) by RT-qPCR (**Figure 3A**), Western blotting (**Figure 3B** and** C**), and flow cytometry (**Figure 3D**). These results prompted us to analyze their sphere-forming capacity, which is a classical functional assessment of their stemness and tumorigenicity. We found that *SETD1B* KD greatly inhibited the growth capacity of stem cell spheres formed by HCCLM3 and HepG2 LCSCs resulting in significantly smaller sphere sizes (**Figure 3E** and** F**; **Figure S3A** and** B**). Altogether, these findings suggest that SETD1B is likely involved in the maintenance of stemness in LCSCs, thereby playing a regulatory role in the development of HCC.

### SETD1B maintains cell stemness and adhesion

**Figure 4:**
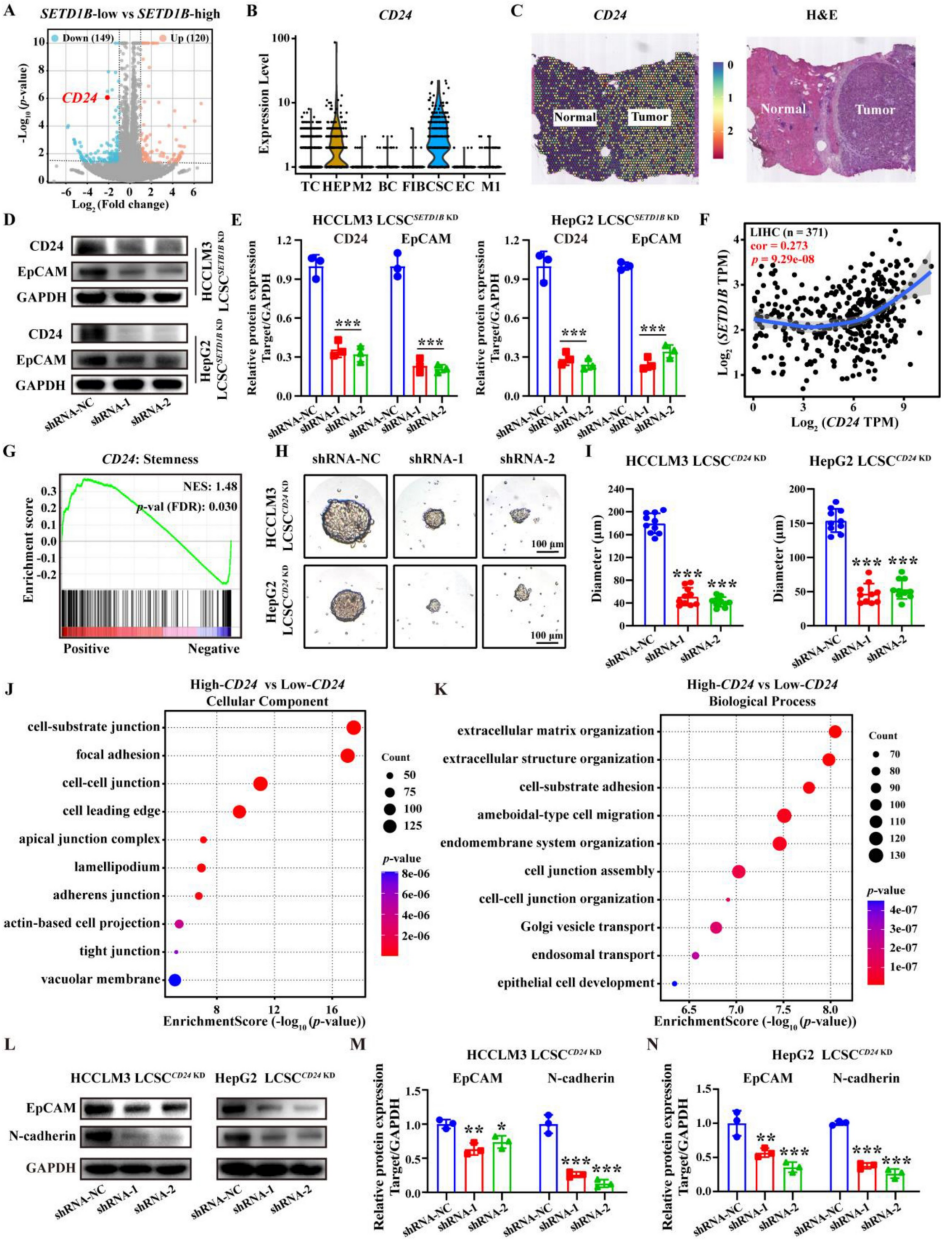
***SETD1B* KD down-regulates LCSC stemness via surface CD24 glycoprotein. (A)** Volcano plot for* SETD1B*-low vs *SETD1B*-high. **(B)** Single-cell RNA sequencing (GSE125449) analysis of* CD24* gene expression across distinct cellular clusters. **(C)** Spatial transcriptomics (HRA000437) analysis of *CD24* expression in liver tumor-adjacent tissue and HCC tissue. **(D) (E)** The proteins expression and quantification of CD24 and EpCAM after *SETD1B* KD. (F) Correlation of *SETD1B* with *CD24* gene expression. **(G)** Enrichment of* CD24* with stemness signaling pathway. **(H)** Spheroid formation ability of HCCLM3 and HepG2 LCSCs after *CD24* KD. **(I)** Quantification of spheroid formation ability and clone number of HCCLM3 and HepG2 LCSCs after *CD24* KD. **(J) (K)** GO enrichment (Cellular Component and Biological Process) for high and low gene expression of *CD24*. **(L)-(N)** Proteins expression and grayscale value statistics of EpCAM and N-cadherin after *CD24* KD. ** p < 0.01, *** p < 0.001.

To investigate how SETD1B mediates its function in cellular stemness, we subjected sorafenib-resistant HCCLM3 cells with and without *SETD1B* KD to bulk RNA sequencing (bulk RNA-seq). Differential gene expression analyses identified numerous significantly changed genes and pathways related to cell stemness (data not shown), and several such genes exhibiting substantial down-regulation were further examined through scRNA-seq of HCC specimen. The results showed that *CEACAM6* and *CD24* were specifically highly expressed in cancer stem cell (CSC) (**Figure 4A** and **B**; **Figure S4A**). Analysis of pan-cancer expression for *CEACAM6* and *CD24* revealed that both levels were significantly altered in a number of cancer tissues compared to their respective normal counterparts, such as breast invasive carcinoma (BRCA), lung adenocarcinoma (LUAD), and thyroid carcinoma (THCA) (**Figure S4C**) 23-25. However, in HCC, *CEACAM6* expression was not prominent, while *CD24* expression was significantly higher compared to normal liver tissues, spatial transcriptomics (HRA000437) of patient tumor specimens corroborated the findings (**Figure 4C**; **Figure S4B** and** C**). Moreover, *CEACAM6* expression showed no significant correlation with survival in HCC patients, whereas low *CD24* expression was associated with a longer overall survival in patients with HCC (**Figure S4D**). These findings indicate that CD24 serves as a potential prognostic biomarker for HCC, whereas CEACAM6 does not exhibit the same predictive value 26. RT-qPCR and Western blotting further confirmed the reduction of CD24 mRNA and protein levels in both HCCLM3 and HepG2 LCSCs upon *SETD1B* KD, respectively (**Figure 4D** and **E**; **Figure S4E**). This increase *CD24* expression was also found to be positively correlated with *SETD1B* expression, suggesting that CD24 is a downstream effector of SETD1B (**Figure 4F**). Notably, we found *CD24* expression was enriched with stemness marker (**Figure 4G**). Indeed, KD of *CD24* in HCCLM3 and HepG2 LCSCs significantly reduced the number of stem cell sphere colonies and resulted in dramatically reduced sphere sizes (**Figure 4H** and** I**; **Figure S4F** and **G**). It also required more stem cells to form a viable sphere (**Figure S4I** and** J**), suggesting reduced tumorigenicity in these cells. To decipher the downstream pathways regulated by CD24, we performed gene ontology (GO) analyses with CD24 as the only variable using a publicly available set of bulk RNA-seq data (GSE141503), which resulted in significant enrichment in various adhesion molecule signaling pathways (**Figure 4J** and **K**). As N-cadherin is the key molecule that govern cell-cell adhesion, we further assessed their changes in HCCLM3 and HepG2 LCSCs after *CD24* KD and found N-cadherin to be significantly down-regulated by Western blotting (**Figure 4L**-**N**; **Figure S4H**). Collectively, these findings demonstrate that SETD1B modulates the expression of CD24, which in turn suppresses the expression of the adhesion molecule N-cadherin, leading to the suppression of stem cell characteristics in LCSCs.

### Identification of transcription factors that modulate the expression of SETD1B

**Figure 5:**
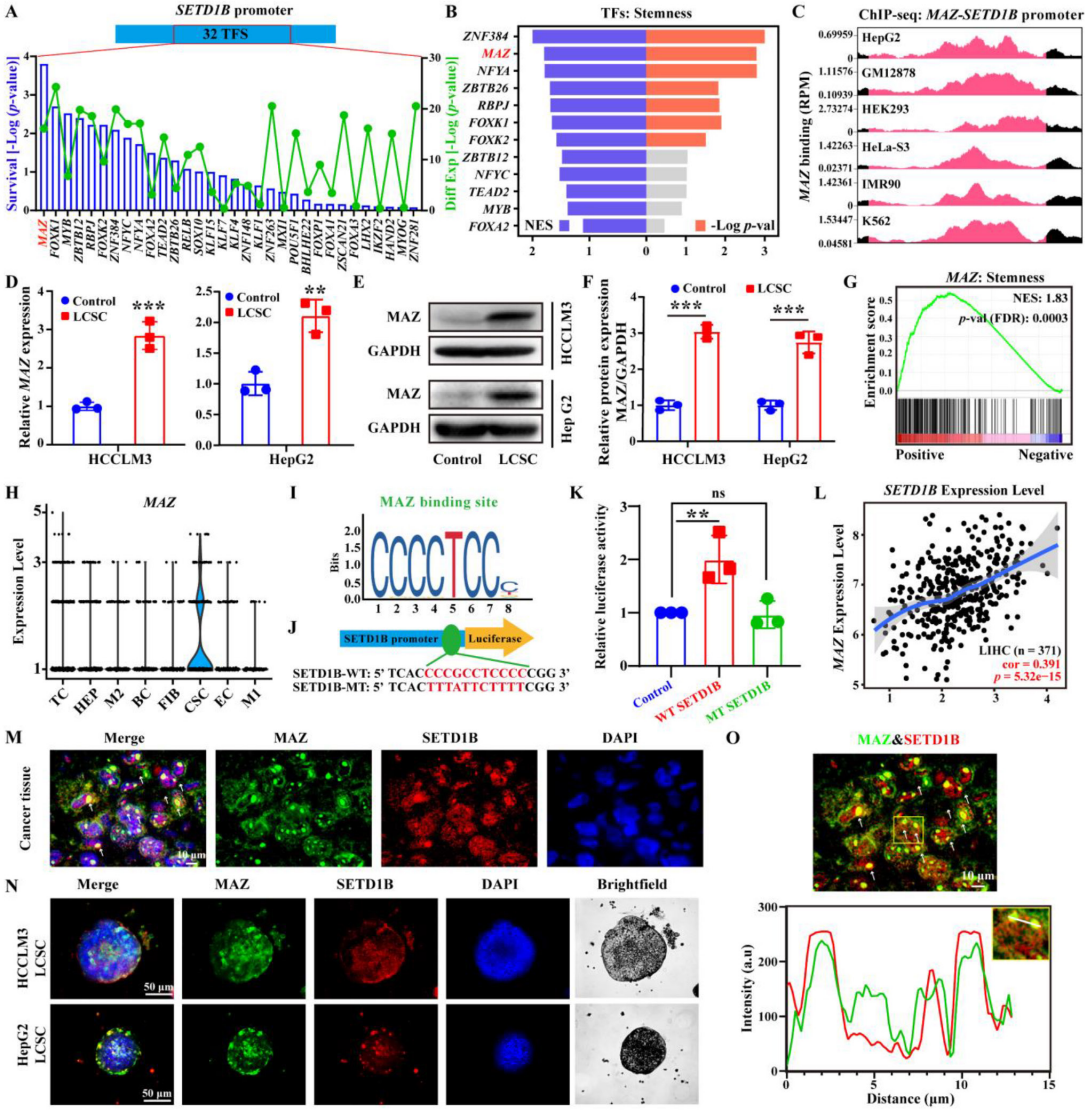
** MAZ transcriptionally regulates SETD1B expression in LCSCs. (A)** Screening of transcription factors that can regulate *SETD1B* using the JASPER database. **(B)** NES and *p*-value of transcription factors associated with stemness enrichment. **(C)** *SETD1B* enrichment at *MAZ* binding sites (reads per million). **(D)** mRNA expression of *MAZ* in HCCLM3/HepG2 cells and LCSCs. **(E)** Expression of MAZ proteins in HCCLM3/HepG2 cells and LCSCs. **(F)** Quantification of MAZ proteins in HCCLM3/HepG2 cells and LCSCs. **(G)** Enrichment of *MAZ* in stemness signaling pathway. **(H)** Expression *MAZ* in single-cell data (GSE125449). **(I)** The JASPAR database predicted the binding motif of *MAZ*. **(J)** Schematic diagram of dual flourescenece reporter plasmid mutation sites for *MAZ* and *SETD1B*. **(K)** Effects of *MAZ* on transcriptional activity in wild-type *SETD1B* versus mutant-type *SETD1B*. **(L)** Correlation between *SETD1B* and *MAZ* expression levels. **(M)-(O)** IF staining of MAZ and SETD1B in HCC tissue and LCSCs. ** p < 0.01, *** p < 0.001, ns: no significant difference.

Next, we sought to elucidate the regulatory mechanism of SETD1B expression in LCSCs. As such, we adopted an unbiased comprehensive approach to identify upstream regulators. We first retrieved the promoter sequence of *SETD1B* and screened for potential transcription factors (TF) using the JASPAR database (https://jaspar.elixir.no). This yielded 32 candidate TFs, of which MAZ (MYC Associated Zinc Finger Protein) was ranked among the highest according to the probability of survival in liver cancer patients (**Figure 5A**; **Figure S5A** and** B**). GSEA enrichment analysis revealed that *ZNF384* and *MAZ* were the top-ranked transcription factors associated with cellular stemness (**Figure 5B**). To this end, we obtained existing datasets for chromatin immunoprecipitation from several cell lines including HepG2 cells. Our analyses of publicly available ChIP-seq datasets (http://cistrome.org/db/) revealed that, compared to *ZNF384*, there were significantly more reads in the *SETD1B* promoter region with *MAZ* (**Figure 5C**; **Figure S5C**). Thus, we selected MAZ for further investigation. In accordance with our *in silico* analyses, we confirmed that MAZ mRNA and protein expressions level were both significantly increased in our established LCSCs compared to their parental controls (**Figure 5D**-**F**). GSEA analysis revealed a positive correlation between *MAZ* expression and pathways governing cell stemness (NES: 1.83) (**Figure 5G**). Our IF analyses demonstrated a significant increase in MAZ and c-MYC expression in both human and SD rat HCC specimens in contrast to their normal tissue counterparts (**Figure S5D** and **E**). Furthermore, analysis of a publicly available scRNA-seq data set on HCC (GSE125449) showed that *MAZ* was specifically expressed in the CSC population (**Figure 5H**; **Figure S5F**-**I**). Thus, we investigated whether MAZ drives SETD1B expression in LCSCs. To this end, through the dual-luciferase reporter assay, we provided evidence that MAZ functions as a transcription factor of SETD1B and is capable of activating SETD1B expression (**Figure 5I**-**K**). Moreover, MAZ was highly expressed in LIHC specimens, and its expression was significantly positively correlated with that of SETD1B (**Figure 5L**; **Figure S5J**). We further investigated the expression pattern of MAZ in HCC tissues and our established LCSCs. As shown in **Figure 5M-O**, IF analysis indicated that both MAZ and SETD1B were co-expressed in nuclei of the HCC tissues and LCSCs. Together, these results show that MAZ is highly expressed in HCC, particularly LCSC, which may potentially drive SETD1B expression that maintains its stemness.

### The transcription factor MAZ regulates the expression of SETD1B

**Figure 6:**
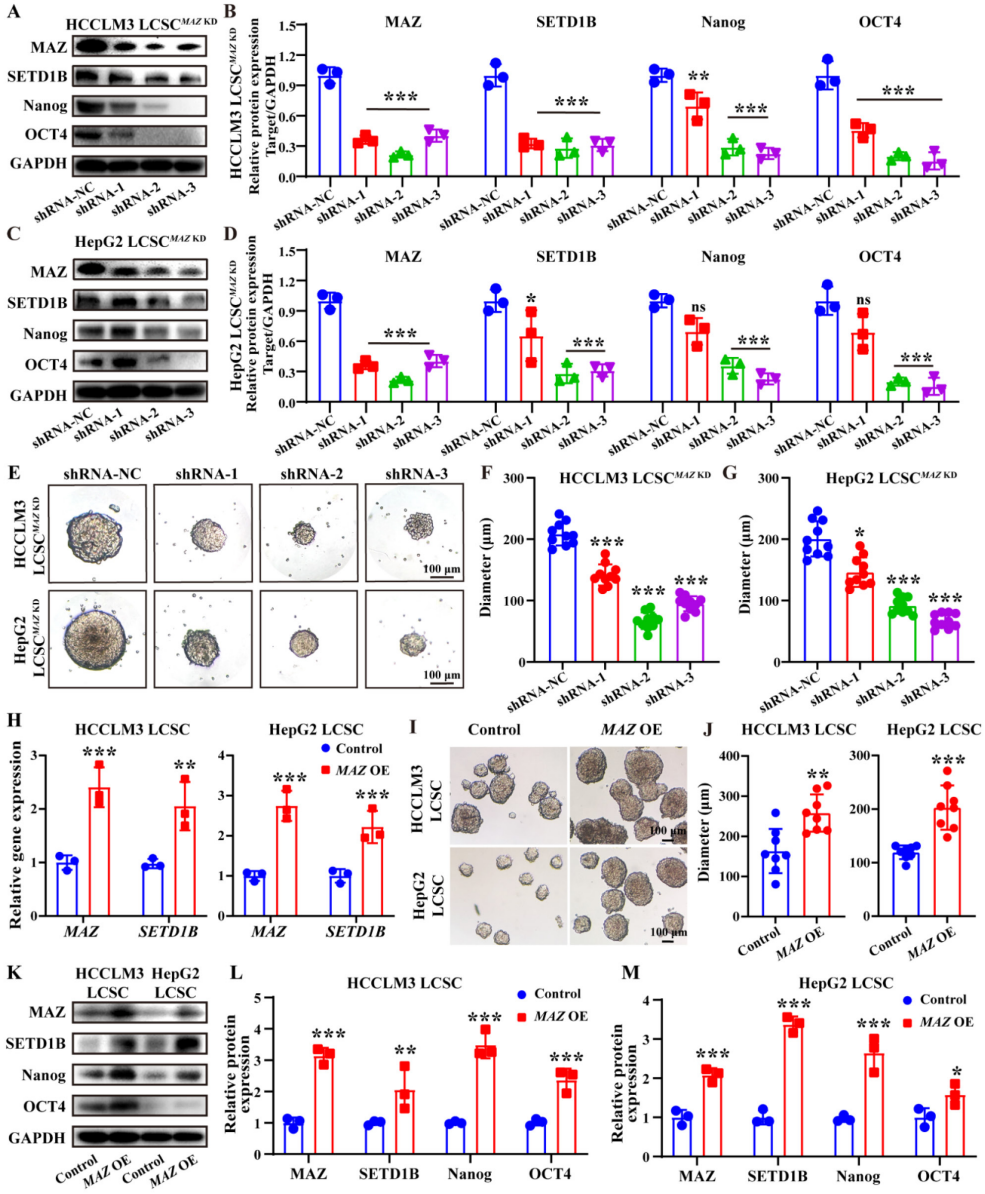
**MAZ induces liver cancer stemness via SETD1B. (A) (B)** The proteins expression and grayscale value statistics of MAZ, SETD1B, Nanog and OCT4 in HCCLM3 LCSCs after *MAZ* KD. **(C) (D)** The proteins expression and quantification of MAZ, SETD1B, Nanog and OCT4 in HepG2 LCSCs after *MAZ* KD. **(E)** Spheroid formation ability of HCCLM3 and HepG2 LCSCs after *MAZ* KD. **(F)**,** (G)** Quantification of the diameters of HCCLM3 and HepG2 LCSCs spheroids after *MAZ* KD. **(H)** Expression of *MAZ* and* SETD1B* gene in HCCLM3 and HepG2 LCSCs after *MAZ* OE. **(I)** Spheroid formation ability of HCCLM3 and HepG2 LCSCs after *MAZ* OE. **(J)** Quantification of the diameters of HCCLM3 and HepG2 LCSCs spheroids after *MAZ* OE. **(K-M)** The proteins expression and grayscale value statistics of MAZ, SETD1B, Nanog and OCT4 in HCCLM3 and HepG2 LCSCs after *MAZ* OE. * p < 0.05, ** p < 0.01, *** p < 0.001, ns: no significant difference.

To ascertain the regulatory role of MAZ in SETD1B expression, we used a similar approach, delivered MAZ-targeting shRNA and *MAZ* CDS into these LCSCs using lentiviral vectors, and then assessed the alteration of their stemness. Following *MAZ* KD, we observed a concomitant down-regulation of SETD1B at both mRNA and protein levels as well as the stem cell markers Nanog and OCT4 in both HCCLM3 and HepG2 LCSCs (**Figure 6A**-**D**; **Figure S6A** and **B**). Similar to our previous findings, KD of *MAZ* also led to a significant decrease of the stem cell sphere growth capacities of HCCLM3 and HepG2 LCSCs (**Figure 6E-G**; **Figure S6C** and **D**). Interestingly, *MAZ* overexpress led to a concomitant upregulation of SETD1B at both transcriptional (**Figure 6H**) and translational levels (**Figure 6K-M**), and LCSCs displayed more pronounced stem-like characteristics (**Figure 6I** and **J**). Taken together, these data indicate that MAZ regulates SETD1B expression in LCSCs which in turn influences their stemness.

### Triptolide targeting the SETD1B protein inhibits the stemness of stem cells, inhibiting tumor growth

**Figure 7:**
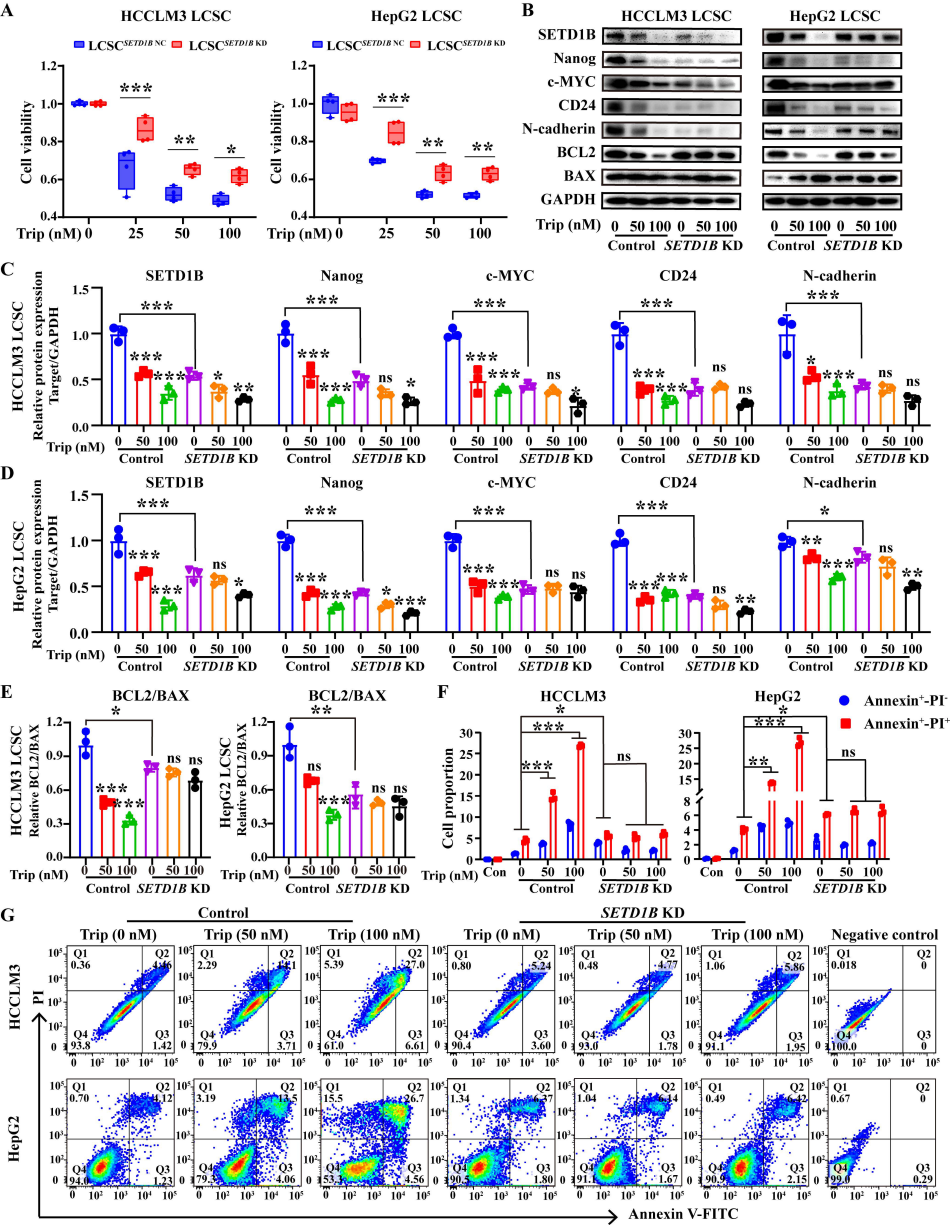
** SETD1B-expressing LCSCs are sensitive to triptolide treatment. (A)** Detection of cell viability for Trip (0, 25, 50, 100 nM) treatment on HCCLM3 and HepG2 LCSCs. **(B)** The proteins expression of SETD1B, CD24, stemness markers (Nanog and c-MYC), adhesion molecules marker (N-cadherin) and BCL2/BAX after Trip treatment on HCCLM3 and HepG2 LCSCs. **(C)-(E)** Quantification of the grayscale values of proteins after Trip treatment on HCCLM3 and HepG2 LCSCs. **(F) (G)** Detection and statistical analysis of cell apoptosis after Trip treatment on HCCLM3 and HepG2 LCSCs by FC. * p < 0.05, ** p < 0.01, *** p < 0.001, ns: no significant difference.

Thus far, our results have demonstrated the critical role of SETD1B in defining stem-like characteristics in LCSCs and tumor progression, suggesting potential therapeutic benefits in targeting this molecule. Unfortunately, there are no available FDA-approved drug that can target SETD1B; therefore, we surveyed a bank of small natural molecular drugs that not only exhibit considerable promise in disease management, but also show potential for targeting SETD1B based on our previous unpublished data, such as eganelisib, dactolisib, curcumin, trametinib, and triptolide 27-31. Molecular docking showed that triptolide (Trip), a natural product derived from *Tripterygium wilfordii*, exhibited the strongest binding affinity for SETD1B, forming hydrogen bonds at multiple sites to establish a stable structure (**Figure S7A** and** B**). Thus, it was chosen for downstream investigation. CCK8 assays revealed that the IC_50_ values of Trip on HCCLM3 and HepG2 LCSCs were 83.93 nM and 80.20 nM, which indicated remarkable drug sensitivity of these cells to this compound (**Figure S7C**). In addition, *SETD1B* KD significantly attenuated the inhibitory effect of Trip on cell viability (**Figure 7A**). To explore whether Trip affected SETD1B expression, we performed analyses on Trip-treated LCSCs with and without *SETD1B* KD. Interestingly, Trip treatment led to a dramatic reduction in SETD1B and other stemness, adhesion, and apoptosis markers in parental LCSCs which became unresponsive to Trip-treatment after *SETD1B* KD (**Figure 7B**-**E**). Further assessment with MG132 in HCCLM3 LCSCs revealed that this Trip-induced reduction in SETD1B happened at both transcript and protein levels (**Figure S7D-G**). Moreover, we unveiled a dosage-dependent reduction in stem cell clonal formation and proliferation following Trip treatment, which was also abolished by prior *SETD1B* KD (**Figure S7H**-**K**, **S8A** and **B**). Most notably, the Trip-induced dosage-dependent cell death of both HCCLM3 and HepG2 LCSCs was no longer observed after *SETD1B* KD (**Figure 7F** and **G**). To further evaluate the target specificity of Trip on SETD1B, we characterized the effects of two other widely used SETD1B inhibitors (Chaetocin 19 and UNC0379 32) on our LCSCs. Both inhibitors significantly reduced *SETD1B* expression and produced phenotypic outcomes consistent with the *SETD1B* KD group in our LCSCs. Notably, similar to our previous findings, Trip lost its function when the LCSCs were pretreated with either one of the inhibitory drugs (**Figure S8C**-**H**). These results demonstrated that Trip inhibits cancer stemness by suppressing SETD1B expression.

**Figure 8:**
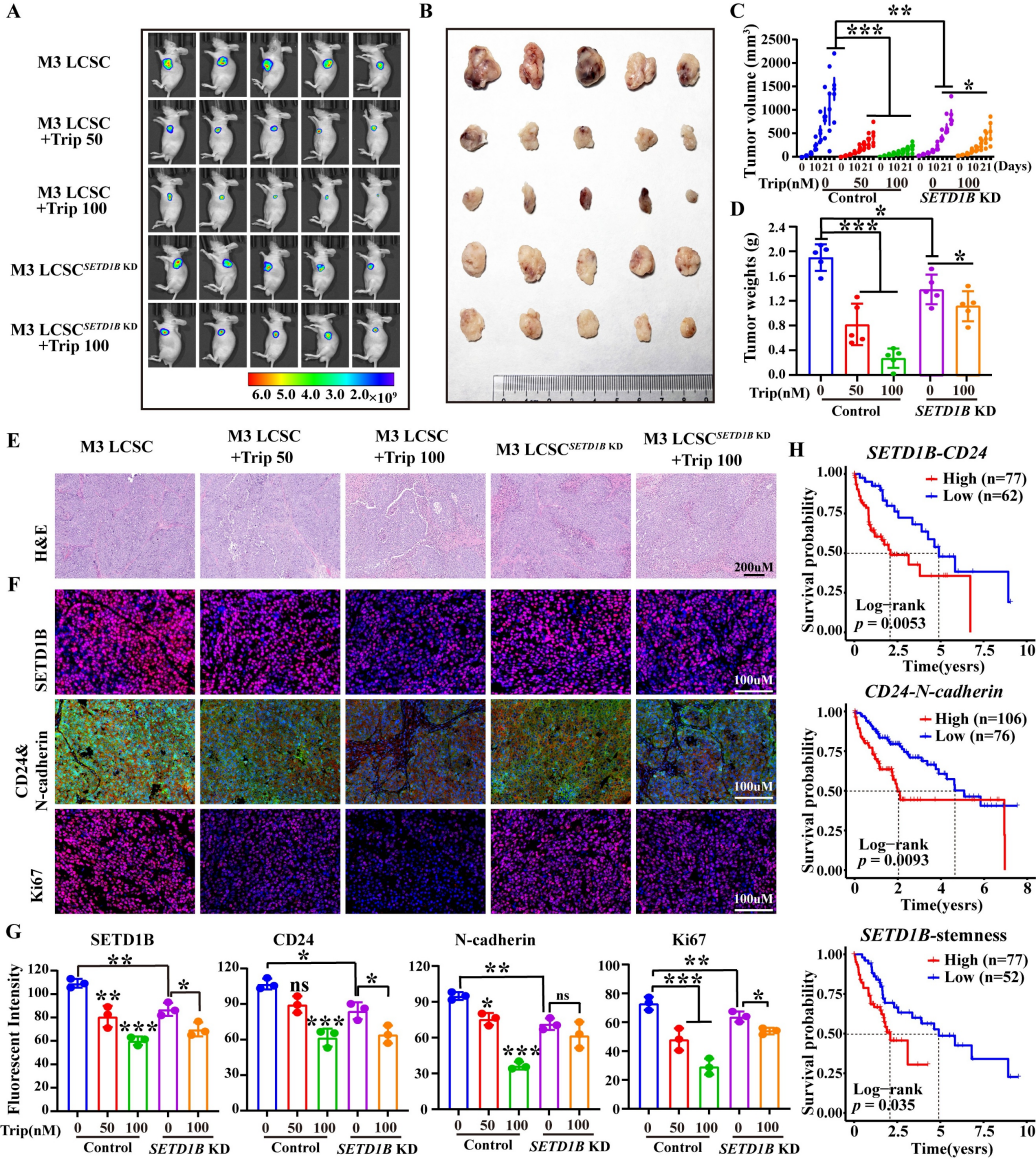
** Triptolide inhibits HCC progression by suppressing SETD1B-driven stemness. (A)*** In vivo* living imaging of Trip's effect on tumor formation in nude mice. **(B)** Impact of Trip on the tumorigenicity of HCCLM3 LCSCs in nude mice. **(C)** Statistical analysis of tumor volumes. **(D)** Statistical analysis of tumor weights. **(E)** Histological staining **(H&E)** analysis of tumor tissues from each group. **(F)** Immunohistochemical staining of tumor tissues with SETD1B, CD24, N-cadherin and Ki67. **(G)** The statistical assessment of the mean fluorescence of SETD1B, CD24, N-cadherin and Ki67 in tumor tissues from each group. **(H)** Correlation between *SETD1B*-*CD24, CD24*-*N-cadherin*, and *SETD1B*-mediated LCSC stemness and poor prognosis in patients with HCC, respectively. * p < 0.05, ** p < 0.01, *** p < 0.001, ns: no significant difference.

Surprisingly, HCC xenografts showed a significant response to Trip treatment at nanomolar ranges in our mouse models (**Figure 8A**-**D**). However, although SETD1B resulted in noticeably smaller tumors, similar to previous observations, these tumors exhibited a substantial decrease in response to Trip treatment even at the highest dosage tested (**Figure 8A**-**D**). Indeed, further pathological analyses showed reduction of SETD1B, CD24, N-cadherin and Ki67 following Trip treatment in the tumor xenografts, but reduction of these proteins was no longer seen with *SETD1B* KD (**Figure 8E**-**G**). Finally, our analysis of public databases revealed that elevated expression and stemness of the genes *SETD1B*, *CD24*, and *N-cadherin* were significantly correlated with poorer prognosis in HCC patients' post-treatment (**Figure 8H**). This correlation implies that a SETD1B-driven stemness microenvironment negatively affects patient survival rates. Altogether, we identified a promising therapeutic agent, Trip, that inhibited tumor progression in our preclinical mouse models of HCC by targeting SETD1B to suppress the stemness of LCSC. These findings underscore the clinical significance of targeting SETD1B with Trip, potentially enhancing the therapeutic outcomes.

## Discussion

Cancer stem cells (CSCs) are characterized by their ability to self-renew, drive tumorigenesis, and confer tumor heterogeneity, which poses a significant challenge for cancer treatment. Therefore, understanding the molecular basis behind their stemness is key to the advancement of current therapeutics for HCC 33, 34. Here, we report that the methyltransferase SETD1B plays a crucial role in the development of HCC by contributing to the stemness of liver cancer stem cells (LCSCs). Our research revealed that SETD1B expression is abnormally high in HCC, especially in the LCSC subpopulations, which maintains their stem cell-like characteristics. SETD1B levels are driven by the transcription factor MAZ, which conveys its function via the surface glycoprotein, CD24, to modulate cellular adhesion and tumorigenicity. Moreover, SETD1B can be targeted by triptolide for degradation, leading to the eradication of LCSCs and a dramatic reduction in HCC. Our study not only uncovered an important contributor of LCSCs in HCC but also provided new perspectives for potential therapeutic approaches for this devastating disease.

As a member of the human SET1 family, SETD1B is capable of catalyzing the methylation of histone H3 at lysine 4 (H3K4) 35, and abnormally elevated levels of SETD1B expression correlate with poor prognostic outcomes in HCC 36. Similarly, Chen et al. found that high expression levels of SETD1B were significantly associated with larger tumor size (*p* < 0.05), advanced clinical tumor stages (*p* < 0.01), and the presence of liver cirrhosis (*p* < 0.05) 36. Using multiple bioinformatics approaches, our research further revealed that SETD1B expression is elevated in HCC stem cells compared to HCC cells, potentially accounting for the enhanced tumorigenic potential observed in HCC stem cells 33, 34. This is reflected by the association of SETD1B levels with the global stemness signature as KD of *SET1DB* simultaneously altered the expression of characteristic stemness marker genes, suggesting that SETD1B is capable of systematically reprogramming HCC cells into pernicious cancer stem cells. A previous study supports this hypothesis by showing that SETD1B plays an indispensable role in the early stages of organogenesis and is crucial for the maintenance of long-term hematopoietic stem cells 21, 22. Once formed, cancer stem cells maintain their self-renewal capacity within the body through symmetric and asymmetric division 37. Typically, the stronger the pluripotency of stem cells, the less they rely on anchorage for growth, which leads to higher oncogenicity 38. In our research, we demonstrated that prolonged cultivation of HCC cells can trigger the expression of characteristic stem cell-specific genes, indicating their reprogramming towards LCSC-like cells. Interestingly, *SETD1B* expression was also highly elevated during this reprogramming process, which further implicates it in HCC and LCSCs. Moreover, KD of *SETD1B* significantly inhibited anchorage-dependent growth and the size and number of stem cell spheres, leading to decreased cell stemness and tumorigenic potential. These findings, suggest that SETD1B is an important contributor of LCSCs stemness and that disrupting SETD1B function can be beneficial for patients with HCC. As SETD1B has methyltransferase function, which can potentially exert significant epigenetic changes that influence numerous stemness-related genes, it is perhaps not surprising that functional perturbation of SETD1B alone can have a profound effect on LCSCs 22, 39. Hence, our results not only corroborate the observations reported in the previous article but also further demonstrate the role of SETD1B in HCC by offering a potential explanation at the cellular and molecular level for its association with poor prognosis.

Given the critical role of SETD1B in enhancing HCC stemness and progression, we explored its upstream regulatory mechanism. Our study revealed that MAZ was highly expressed in HCC and acted as a driver of *SETD1B* expression. Interestingly, we found that down-regulation of SETD1B in LCSC leads to reduced cell stemness due to a concomitant decrease in c-MYC levels. Traditionally, MAZ is known as an important regulator of *c-MYC* expression, which directly influences the self-renewal and maintenance of pluripotency in stem cells 40, 41. Our results provided further evidence that MAZ-mediated regulation of c-MYC and changes in cellular stemness may be executed in part by SETD1B. However, MAZ has also been shown to modulate the alternative splicing of the c-MYC gene, which in turn impacts the self-renewal capacity and the maintenance of pluripotency in stem cells 40, 41, and concurrently, it also engages in interactions with CDK6, thereby sustaining the functional integrity of hematopoietic stem cells 42. Therefore, MAZ and SETD1B may synergistically regulate HCC stemness, but further research is needed to determine whether MAZ alone can influence these cellular stemness-related genes alone. However, how does SETD1B influence HCC stemness? The current study offers a plausible mechanism by showing SETD1B-dependent regulation via the CD24 transmembrane glycoprotein. CD24 functions as a modulator of cell migration, invasion, and proliferation 43. At the same time, CD24 is also a marker of stemness as it is highly expressed in transit-amplifying cells 44, 45. We conducted gene ontology (GO) enrichment analysis on RNA sequencing data from samples with high and low expression of CD24. The analysis revealed significant enrichment of cellular components and biological processes related to cell-matrix adhesion, which may be regulated by CD24 through its commonly implicated downstream Hippo or p53 pathways resulting in alteration of adhesion molecules 46, 47. This finding is consistent with previous studies on the maintenance of stem cell characteristics. Given the widespread increase in histone methylation in stem cells 48, 49, SETD1B possesses the capability to catalyze the methylation of histone H3 at lysine 4 (H3K4) 35. We speculate that SETD1B may influence the levels of histone H3K4 trimethylation (H3K4me3) in LCSCs, thereby regulating the expression of CD24. This regulatory effect may further affect the stemness characteristics and spheroid-forming ability of stem cells. However, further analysis of the methylation status is needed to uncover the specific molecular mechanisms by which SETD1B regulates CD24 expression.

The ongoing development of novel targeted therapies has significantly bolstered the survival rates of patients 50. A variety of compounds derived from natural plant sources, recognized for their potential to inhibit liver cancer, have paved the way for innovative therapeutic approaches for tumor management 51, 52. As SETD1B is a critical regulatory factor in HCC progression, targeting this protein for degradation may be a potential therapeutic strategy 53. Consequently, through selective screening of natural products, we discovered that triptolide (Trip) could specifically target SETD1B for degradation and trigger dosage-dependent cell death in LCSCs *in vitro* and *in vivo*. We find that the ability of Trip for targeting SETD1B is likely attributable to the ability of the hydroxyl group in Trip to form a stable hydrogen bond with the carboxylic acid group of arginine at position 185, lysine at position 125 and 133, and asparagine at position 111 within the active site of the SETD1B protein. This interaction suggests the possible formation of a SETD1B-Trip complex, which is then shunted to the proteasome for degradation 54. However, the exact mechanism of this degradation requires further research. More importantly, we observed that Trip significantly suppressed the expression of adhesion molecules and the ability to form spheres in LCSCs, thereby promoting apoptosis in LCSCs. This result suggests that LCSCs enhance their defense mechanisms against external cytotoxic factors by highly expressing adhesion molecules, and the intervention of Trip effectively weakens this defense 55, 56. In addition, by targeting ERCC3 and regulating the c-MYC/miRNA cluster/target gene axis, Trip significantly enhances its ability to induce apoptosis in liver cancer cell lines both *in vitro* and* in vivo* 57. Nevertheless, Trip possesses the capability to not only effectively inhibits liver cancer, but also influences other malignancies, including lung cancer, pancreatic cancer, nerve tumors, and prostate cancer, through various signaling pathways. Specifically, Trip exerts its antitumor effects by modulating critical signaling pathways such as Nrf2-ARE, PI3K/AKT/mTOR, Notch, and β-Catenin/Wnt 57-63. Notably, unlike conventional chemotherapeutic drugs, which are mostly administered intravenously, previous research reports have indicated that Trip is primarily administered orally. Unlike gavage administration, we employed an *ad libitum* feeding method for drug delivery, which has also shown significant inhibitory effects on the liver cancer growth. This discovery offers a potential novel therapeutic strategy and a much less invasive regimen for clinical application.

**Figure 9:**
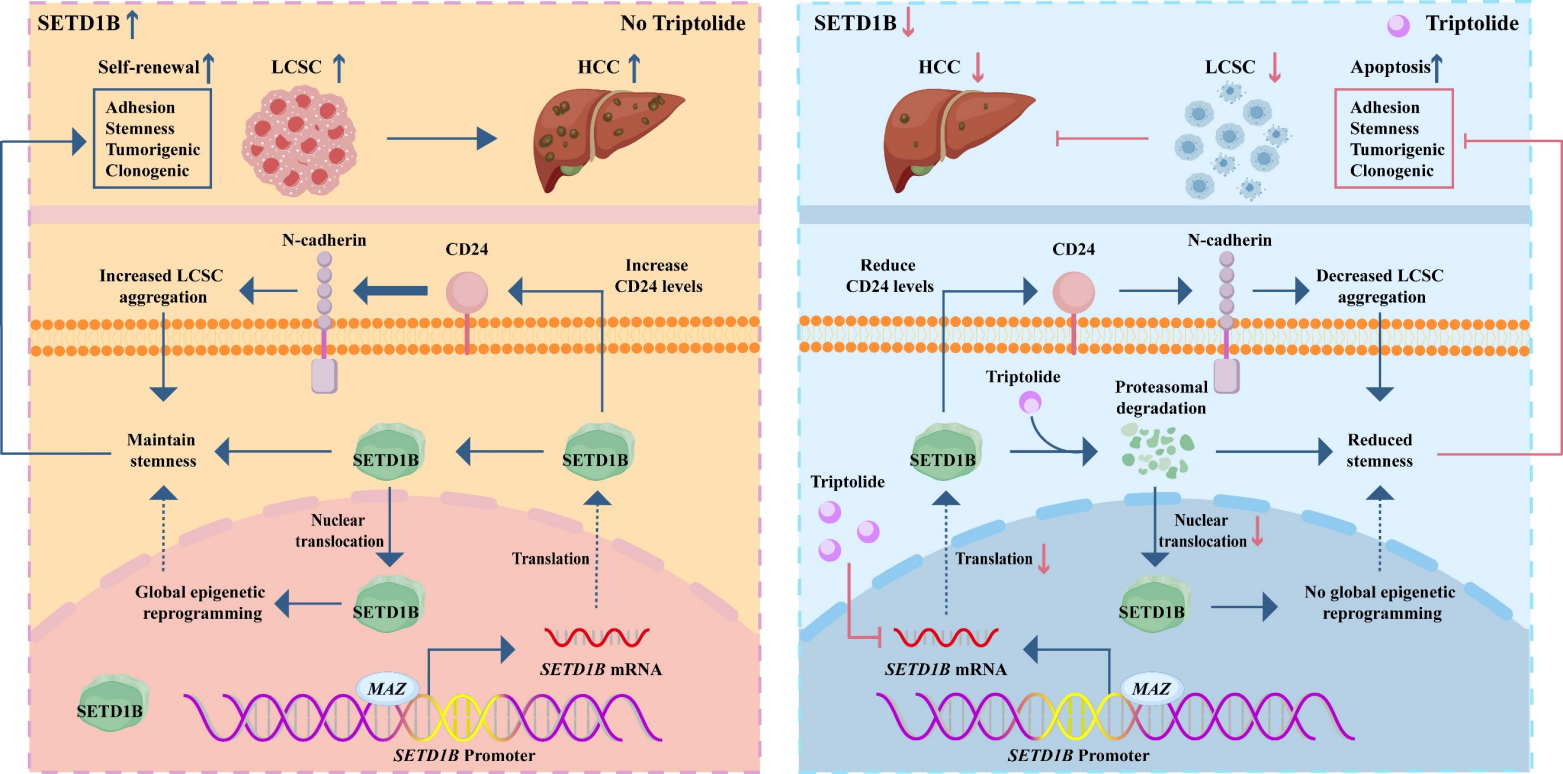
** Graphical summary of the effect and mechanism of triptolide on LCSCs.** The schematic illustrates a pivotal signaling axis, MAZ/SETD1B/CD24, which is essential for maintaining the stem-like properties of LCSCs. In this pathway, the transcription factor MAZ upregulates SETD1B, which promotes tumorigenicity via the downstream effector CD24. Targeting this molecular cascade with the therapeutic agent triptolide effectively eradicates LCSCs, leading to marked inhibition of HCC progression.

In summary, our study uncovered a previously unknown signaling axis that maintains the stemness of LCSCs, wherein SETD1B, under the transcriptional control of MAZ, drives tumorigenicity via CD24. Targeting this molecular cascade by Trip results in the destruction of LCSCs, which in turn drastically hampers HCC progression. These data constitute an important scientific basis for potential therapeutic strategies for HCC (**Figure 9**).

## Materials and methods

### Animal models

The study was approved by the Ethics Committee of the Army Medical University. We established a Sprague Dawley (SD) rat (Vital River, Zhejiang, China) model of primary liver cancer (PLC) according to previous methods 64. Briefly, 5-week-old male Sprague-Dawley (SD) rats were intraperitoneally injected with 1% diethylnitrosamine (DEN) at a dose of 100 mg/kg. The following day, they were administered nitrosomorpholine (NMOR) solution at a concentration of 100 ppm. Liver samples were collected at predetermined time intervals for histological analysis. Nude mice were purchased from the Vital River (Zhejiang, China) and kept in a specific pathogen-free (SPF) environment to reduce the risk of pathogen infection. Two million HCCLM3 LCSCs suspended in PBS were subcutaneously injected into the flank of nude mice. Mice are feed ad libitum. The onset and progression of the tumors were continuously monitored and measured with a digital caliper as early as day 3 following implantation until the tumor size reached the maximum volume allowed by the institution, at which point the mice were sacrificed and the tumor samples were collected for analyses. The volume of the tumor was estimated using the formula volume = (π/6) × length × width^2^ 65.

### Cell lines and culture conditions

Human liver cancer cell (LCCs) lines HCCLM3 and HepG2, were donated by the researchers. To generate LCSCs from the respective cell lines, cells were trypsinized at approximately 90% confluence and dissociated into single cells by gentle pipetting. The cells were pelleted by centrifugation, washed twice with PBS, and resuspended in a complete culture medium to a final concentration of 2000 cells/mL. DMEM/F12 medium supplemented with EGF (20 ng/mL), FGF (10 ng/mL), N2 (1%), B27 (2%) was used to culture liver cancer stem cells (LCSCs) 66. The cell suspension was left undisturbed at 5% CO_2_ and 37°C until the cell spheres reached approximately 100 μm in diameter, at which point the spheres were collected for continuous passage. After three passages, the cells were assessed for stemness and used in subsequent experiments. LCSCs and LCCs lines were cultured in DMEM/F12 and H-DMEM (Gibco, USA) supplemented with 10% FBS (ExCell Bio, China) and 1% penicillin/streptomycin, respectively, and maintained at 37°C and 5% CO_2_ unless indicated otherwise. All cell cultures were tested negative for Mycoplasma.

### Western blotting

Cells were lysed using RIPA buffer with PMSF (1:100, Solarbio, Beijing) according to the previously described method 67. The lysates were separated by SDS-PAGE and transferred onto nitrocellulose membranes. The membranes were then blocked and incubated with various primary antibodies overnight at 4^o^C. For detection, the membranes were incubated with HRP-conjugated secondary antibodies and the chemiluminescence was captured by ECL chemiluminescence imaging system (Bio-OI-X6, China). Densitometrical analysis of the protein bands was performed using ImageJ software. Antibodies and dilution factors are listed in Supplementary Table S1.

### RT-qPCR

The total RNA was isolated from the samples using TRIzol reagent (Invitrogen, Life Technologies, USA). Complementary DNA was synthesized from the RNA using the prime™ RT reagent kit (TaKaRa, Tokyo, Japan) following the manufacturer's instructions. qPCR was carried out using SYBR PremixExTaq™II (TaKaRa, Tokyo, Japan). The relative gene expression levels were calculated using the 2^-ΔΔCt method with *GAPDH* serving as the reference for normalization 68. Primers are listed in Supplementary Table S2.

### ShRNA knockdown

The P3 generation cell spheres, having undergone enrichment and identification, were washed and digested to achieve a cell density of 1.5 × 10^5^ cells/mL. Cells were transiently transfected with Lipofectamine 3000 following the recommended protocols. For stable transfection, the cells were subjected to puromycin selection for an initial 24-hour period. Subsequently, an additional 48 hours of selection was performed to ensure the enrichment of puromycin-resistant cells, indicating successful transfection. The successfully transfected cells were identified and expanded for large-scale culture in preparation for further experimental use. Stable cells were maintained in the complete culture medium with an additional 2 μg/mL puromycin. The shRNA sequences used are listed in Supplementary Table S3.

### Viability assay

The HCCLM3 and HepG2 LCSCs with and without *SETD1B* KD were treated with Trip at 0, 25, 50, and 100 nM for 48 h. The cells were then incubated with CCK8 at 10% (v/v) for 1 h at 5% CO_2_ and 37°C and after which the cell medium was collected for absorbance measurement at 450 nm using a conventional microplate reader (Bio-Rad, Berkeley, CA).

### Flow cytometry

Stem cell spheres were dissociated into single cell suspensions at a density of 5 × 10^6^ cells/mL. The cells were then labeled with anti-CD133-FITC and CD44-FITC (BD, USA) and preserved in cold PBS until they were processed on a BD LSRFortessa^TM^ X-20 flow cytometer (BD Biosciences, USA). The data were analyzed using the FlowJo software. To asses apoptosis, the cells were treated with DMSO or Trip at 37°C for 48 h prior to collection and resuspended in 100 μL of binding buffer (BD, USA). Next, the cells were incubated with FITC-conjugated Annexin V (BD, San Jose, CA, USA) and propidium iodide (PI, BD, USA) at room temperature for 30 min before being analyzed by flow cytometry. The antibodies and dilutions used are listed in Supplementary Table S1.

### Luciferase reporter assay

To construct a luciferase reporter gene vector containing the *SETD1B* promoter, the full-length *SETD1B* promoter containing wild-type or mutant-type was respectively cloned into pGL3-basic vectors (GeneCreate, Wuhan, China). The cells were co-transfected with or without the *MAZ* overexpression (OE) vector using Lipofectamine 3000 (Invitrogen, Carlsbad, CA, USA) in HCCLM3 cells. After 48 h of incubation, the activities of firefly and Renilla luciferase were measured using the Dual Luciferase Reporter Assay Kit (Promega, Madison, WI, USA) 69.

### Bulk RNA sequencing

Total RNA from sorafenib-resistant HCCLM3 with and without *SETD1B* knockdown (KD) was extracted using the TRIzol reagent. RNA was then submitted to Majorbio Bio-pharma Biotechnology Co., Ltd. (Shanghai, China) for high-throughput transcriptome sequencing. RNA-seq data was analyzed using the Majorbio cloud platform (https://www.majorbio.com). To re-analyze the existing dataset, bulk RNA-seq data from breast cancer stem cells with *CD24* KD (GSE141503) were obtained from the NCBI Gene Expression Omnibus (https://www.ncbi.nlm.nih.gov/geo/query/acc.cgi?acc=GSE141503) 70. The visualization of Gene Ontology (GO) enrichment analysis was performed by https://www. bioinformatics.com.cn.

### Single-cell RNA sequencing analysis

Single-cell RNA sequencing (scRNA-seq) data of patient HCC specimens under the accession number GSE125449 was obtained from the GEO database 71. We employed Seurat version 4.3.0, a suite of analytical procedures, for the scRNA-seq data. This workflow encompasses quality control, normalization of the data, feature selection, and execution of both linear and non-linear dimensionality reduction techniques. Furthermore, we performed cell clustering to identify distinct cell clusters and utilized differential expression analysis to identify cluster-specific biomarkers. Finally, we assigned cell type identities to these clusters based on the molecular signatures defined by CellMarker (http://117.50.127.228/CellMarker/) 72. The expression of *MAZ*, *CD24*, and other genes specific to each cell clusters was visualized using R 4.1.0.

### Spheroid formation assay

HCCLM3 and HepG2 LCSCs were seeded in 96-well culture plates at a density of 256 cells/well (n = 5). Cells were treated with chaetocin (100 or 200 nM), UNC0379 (2.5 or 5 μM), or triptolide (50 or 100 nM) for the first 48 hours, and their tumor sphere formation was subsequently assessed by light microscopy after 15 days of culture.

### Statistical analysis

All data are presented as mean ± standard deviation (SD). Statistical analysis was performed using the GraphPad Prism 9 software. Unpaired Student's t-test or ANOVA with *post hoc* Dunnett's or Tukey's test were used for comparison. Statistically significance was set at *p* < 0.05. All experiments were independently repeated at least three times, and a specific number of biological replicates is indicated in each graph.

## Supplementary Material

Supplementary figures and tables.
