# Supplementary material for: Histone Methyltransferase SETD1B Maintains Cancer Stem Cell Niche by Regulating the Crosstalk between CD24 and Surface Adhesion Molecules in Hepatocellular Carcinoma
Source: Int J Biol Sci. 2025 Jul 24;21(11):4798–815. doi: 10.7150/ijbs.112943 (PMC12374818; doi:10.7150/ijbs.112943)
Supplement: Supplementary file 1 — Supplementary figures and tables. [file ijbsv21p4798s1.pdf]

Supplementary Data

Histone Methyltransferase SETD1B Maintains Cancer Stem Cell Niche by Regulating the Crosstalk between CD24 and Surface Adhesion Molecules in Hepatocellular Carcinoma

Yan Gao [1], Wei Zhou [2], Yuehong Gao [3], Shunxi Wang [1], Zhiling Xu [1], Xiao Xiang [1*], Li Yang [1*]

[1]National Innovation and Attracting Talents "111" base, Key Laboratory of Biorheological Science and Technology, Ministry of Education, College of Bioengineering, Chongqing University, Chongqing, 400044, China

[2]Department of Radiation Oncology, Chongqing University Cancer Hospital, Chongqing, China

[3]College of Biology and Environmental Sciences, Jishou University, Jishou, China

[*]Corresponding authors: Li Yang and Xiao Xiang

Corresponding authors Email: yl@cqu.edu.cn, xiaoxiang_bioeng@cqu.edu.cn

Materials and methods

Generation of Sorafenib-resistant Cells

The sorafenib-resistant HCCLM3 cells were generated by a dosage-escalation approach where the cells were first subjected to sorafenib at lower concentrations of 2 μM and 5 μM for one month each and then switched to a higher concentration at 10 μM for another 6 months until the cells entered normal growth kinetics.

Cell Viability Assay

The cell viability was measured by CCK8 assay (Solarbio, CA1210) as described previously [1]. Briefly, the HCCLM3 LCSCs and HepG2 LCSCs in the logarithmic growth phase were seeded into 96-well plates (5000 cells/well) and treated with Trip (0, 10, 25, 50, 100, 200 nM) for 48 h. The cells were then incubated with CCK8 at 10% (v/v) for 1 h at 5% CO_2 and 37°C and after which the cell medium was collected for absorbance measurement at 450 nm using a conventional microplate reader (Bio-Rad, Berkeley, CA).

Immunofluorescence

The tissue sections were deparaffinized with xylene and rehydrated through an ethanol gradient, then subjected to antigen retrieval. The samples were washed three times with PBST and blocked with 1% BSA for 1 h at room temperature. Subsequently, the samples were incubated with various primary antibodies, followed by incubation with fluorescently labeled secondary antibodies. The samples were counterstained by

DAPI before being mounted with an anti-fluorescence quenching agent and stored in a 4°C refrigerator for image acquisition using a confocal microscope (Leica, Germany) equipped with a 20 × objective. Antibodies and dilution factors are listed in Supplementary Table S1.

Clonogenic Assay

The stable LCSCs with different shRNA knockdown were dissociated into single cell suspensions at 5×10^3 cells/mL and plated in 6-well plates at a density of 1000 cells each well. The LCSCs were cultured in DMED medium, then treated with or without Trip for at least 1 week. The colonies were washed with PBS twice and fixed with 4% paraformaldehyde at room temperature for 15 min. The samples were stained with crystal violet for 10 min, washed and air dried at room temperature before being photographed and quantified using a light microscope.

Limiting Dilution assay

HCCLM3 and HepG2 LCSCs cells were seeded into 96-well cell culture plates at densities of 256, 128, 64, 32, 16, 8, 4, 2, and 1 cell per well in replicates of 8. Following a 15-day culture period, the cells were examined microscopically to assess the tumor sphere formation. The number of cell spheres formed in each statistical group was counted and measured, and the frequency of sphere formation was calculated using the ELDA online tool (https://bioinf.wehi.edu.au/software/elda/) as previously described [2].

Gene Set Enrichment Analysis (GSEA)

The gene expression data pertaining to liver cancer was obtained from The Cancer Genome Atlas (TCGA) and analyzed using the Gene Set Enrichment Analysis (GSEA) software (4.3.2). We separated the expression levels of *SETD1B*, *MAZ* and *CD24* by categorizing them into high and low expression groups according to the medium values. The gene matrix was subjected to analyses by GSEA, which produced enrichment scores (ES) and *p*-values for *SETD1B*, *MAZ* and *CD24*, within a pre-defined set of genes involved in cell stemness.

Spatial Transcriptomics

Spatial transcriptome sequencing data from the Genome Sequence Archive (GSA-Human: HRA000437) were accessed via the National Genomics Data Center at https://ngdc.cncb.ac.cn/search/specific?db=hra&q=HRA000437 [3]. The analyses were performed using Seurat version 4.3.0 as described previously. The spatial expression patterns of *SETD1B*, *MAZ*, and *CD24* across various tissue regions were visualized by R 4.1.0.

Molecular Docking

Molecular docking studies between small molecule drugs and SETD1B (PDB DOI: https://doi.org/10.2210/pdb8ILZ/pdb) were conducted using AutoDock software. The AutoGrid component of AutoDock was utilized to compute the grid map necessary for

docking simulations. The best conformations were searched using a genetic algorithm with local search (GA-LS) method. Default docking parameters were employed, and a total of 50 independent docking runs were performed using the AutoDock Tool Kit software. For molecular graphics and visualization, PyMOL package were used.

References

1. Gao Y, Wang S, He L, Wang C, Yang L. Alpinetin Protects Chondrocytes and Exhibits Anti-Inflammatory Effects via the NF-kappaB/ERK Pathway for Alleviating Osteoarthritis. Inflammation. 2020; 43: 1742-50.
2. Hu Y, Smyth GK. ELDA: extreme limiting dilution analysis for comparing depleted and enriched populations in stem cell and other assays. J Immunol Methods. 2009; 347: 70-8.
3. Wu R, Guo W, Qiu X, Wang S, Sui C, Lian Q, et al. Comprehensive analysis of spatial architecture in primary liver cancer. Sci Adv. 2021; 7: eabg3750.

Supplementary Figure

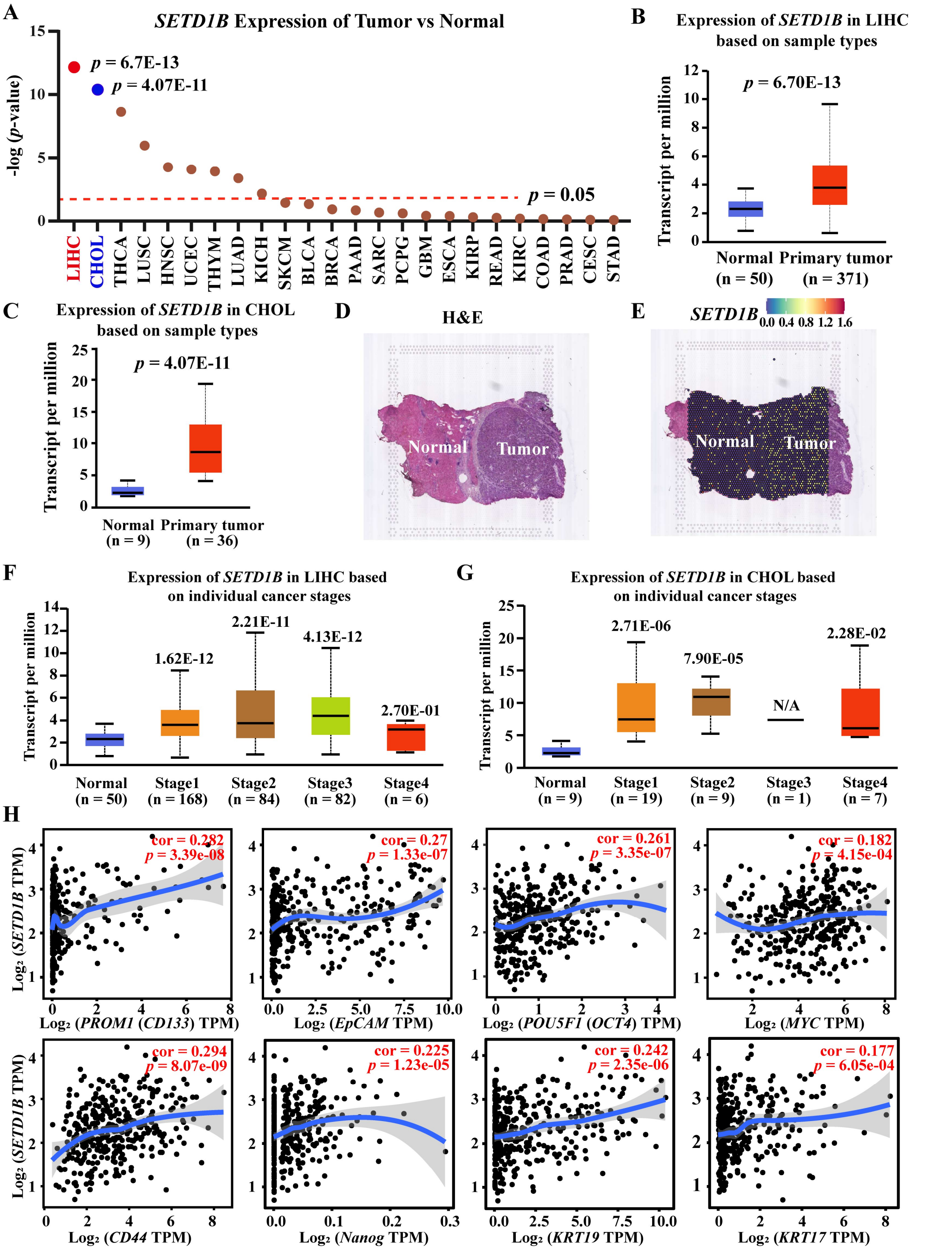

Supplementary Figure S1. SETD1B is highly expressed in hepatocellular carcinoma and cholangiocarcinoma.

(A) *P*-value ranking of *SETD1B* expression difference in normal and cancerous tissues

across various cancer types. (B) (C) Expression levels of *SETD1B* in normal and tumor tissues (LIHC, CHOL). (D) (E) Spatial transcriptomics (HRA000437) analysis of *SETD1B* expression in liver tumor-adjacent tissue and HCC tissue. (F) (G) Expression levels of *SETD1B* in tumor tissues (LIHC, CHOL) on individual cancer stages. (H) Correlation analysis of stemness-related markers *PROM1* (*CD133*), *EpCAM, POU5F1* (*OCT4*)*, MYC, CD44*, *Nanog*, *KRT19*, and *KRT17* with *SETD1B* gene expression.

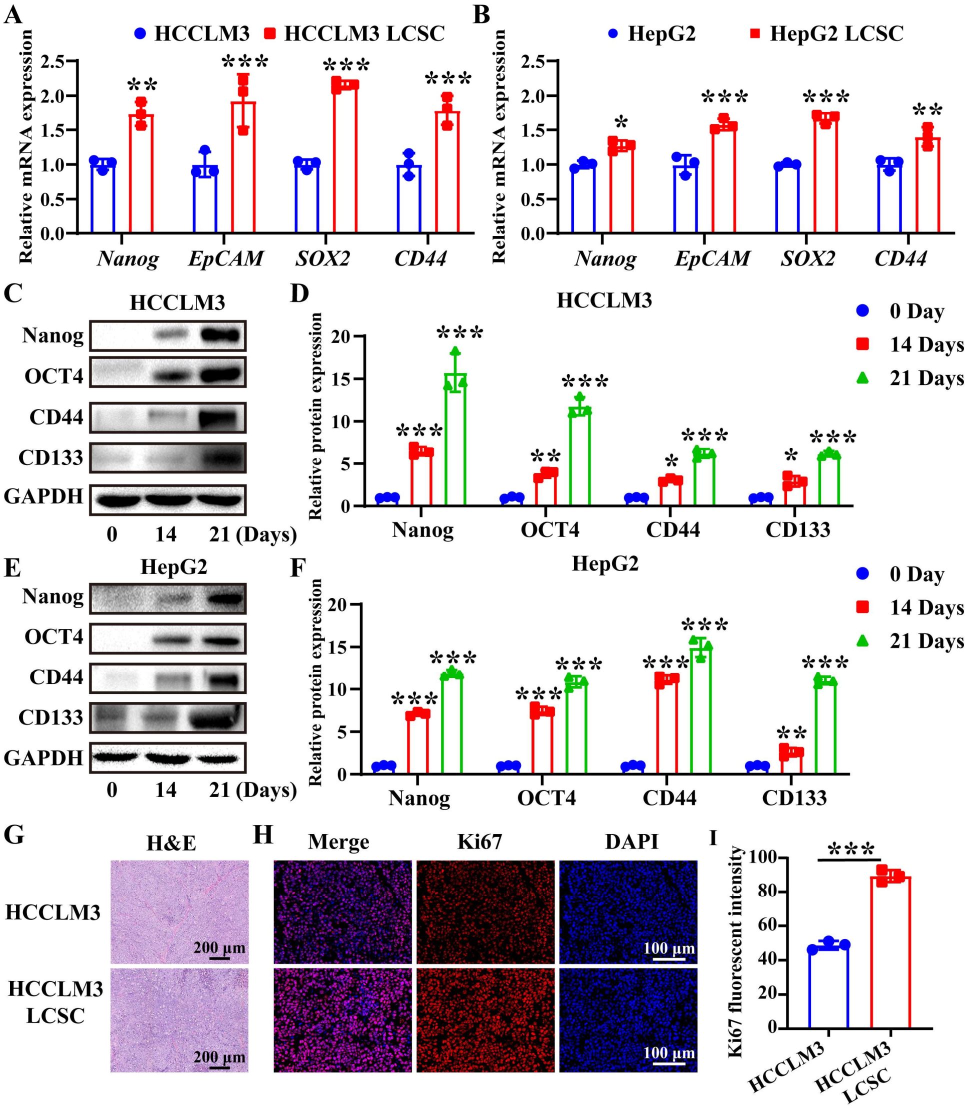

Supplementary Figure S2. Generation and validation of liver cancer stem cells.

(A) Genes expression of stemness markers (*Nanog*, *EpCAM, SOX2*, *CD44*) in HCCLM3 cell and HCCLM3 LCSC. (B) Genes expression of stemness markers (*Nanog*, *EpCAM, SOX2*, *CD44*) in HepG2 cell and LCSC. (C) (E) Proteins expression of stemness markers (Nanog, OCT4, CD44, CD133) in HCCLM3/HepG2 cells and LCSCs. (D) (F) Quantitative image analysis of proteins expression. (G) Histological staining (H&E) analysis of tumor tissues from HCCLM3 cell and HCCLM3 LCSC. (H) Immunohistochemical staining of tumor tissues with Ki67. (I) The statistical assessment of the mean fluorescence of Ki67 in tumor tissues from HCCLM3 cell and

HCCLM3 LCSC. * $p < 0.05$, ** $p < 0.01$, *** $p < 0.001$.

A

HCCLM3 LCSC $^{SETD1B\ KD}$											
Frequency of tumor-sphere formation	Number of cell seeded each well									Stem cell frequence	
Dose	1	2	4	8	16	32	64	128	256	Estimate	Upper and lower limmits
sh*SETD1B* -NC	4	5	5	7	7	8	8	8	8	1:3.7	1:2.30-1:5.96
sh*SETD1B* -1	2	3	4	5	6	6	7	8	8	1:13.1	1:8.30-1:20.70
sh*SETD1B* -2	3	3	3	4	6	6	8	8	8	1:11.1	1:7.02-1:17.56
sh*SETD1B* -3	2	3	4	4	5	6	7	8	8	1:14.9	1:9.42-1:23.46

B

HepG2 LCSC $^{SETD1B\ KD}$											
Frequency of tumor-sphere formation	Number of cell seeded each well									Stem cell frequence	
Dose	1	2	4	8	16	32	64	128	256	Estimate	Upper and lower limmits
sh*SETD1B* -NC	3	3	4	6	7	8	8	8	8	1:5.62	1:3.52-1:8.96
sh*SETD1B* -1	1	3	3	4	4	5	7	7	8	1:14.23	1:9.01-1:22.46
sh*SETD1B* -2	2	2	3	4	5	5	7	8	8	1:13.11	1:8.3-1:20.7
sh*SETD1B* -3	2	3	4	5	6	6	7	8	8	1:18.35	1:11.64-1:28.93

Supplementary Figure S3. *SETD1B* KD inhibits tumor sphere formation.

(A, B) Limited dilution analysis for detecting spheroid formation frequency in HCCLM and HepG2 LCSCs after *SETD1B* KD for 15 days.

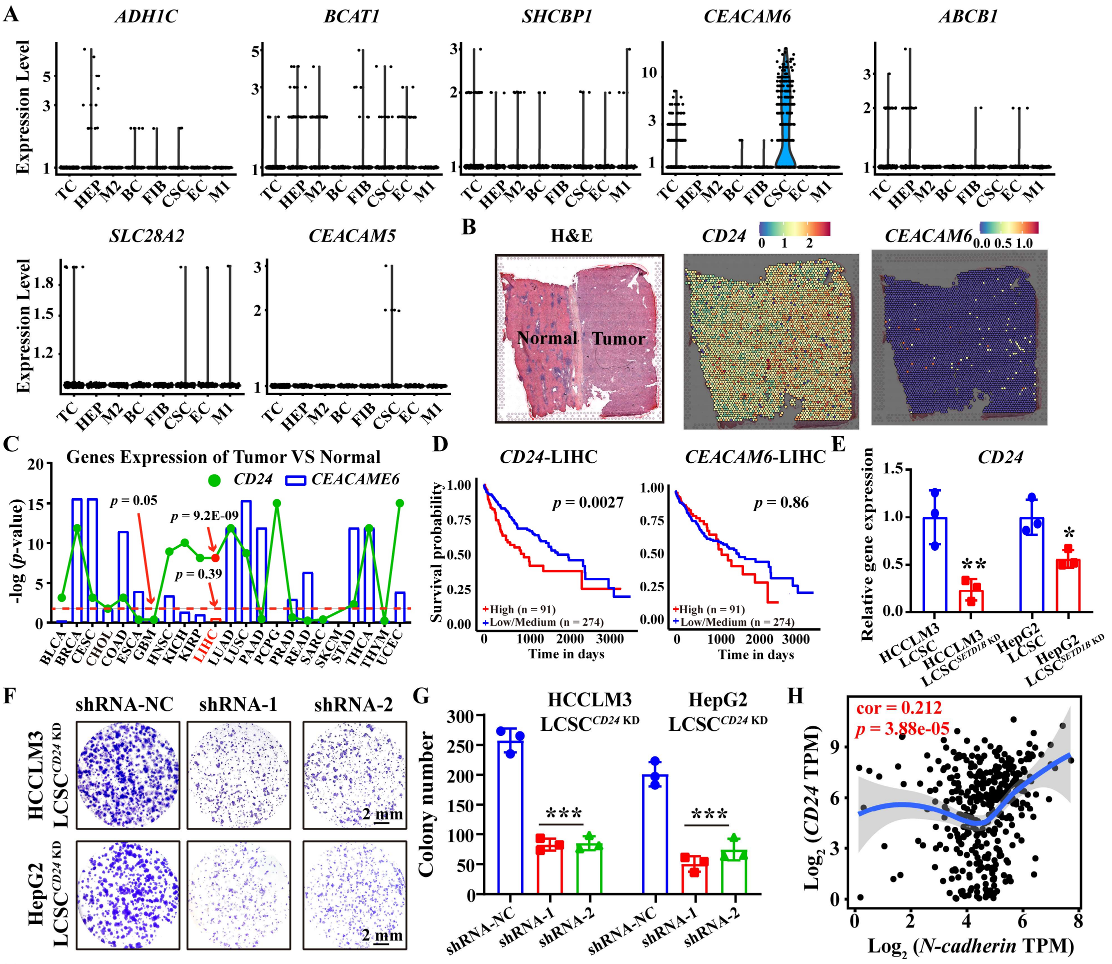

I

HCCLM3 LCSC$^{CD24\ KD}$											
Frequency of tumor-sphere formation	Number of cell seeded each well									Stem cell frequence	
Dose	1	2	4	8	16	32	64	128	256	Estimate	Upper and lower limmits
sh*CD24*-NC	2	3	5	7	7	8	8	8	8	1 : 4.74	1 : 2.96-1 : 7.59
sh*CD24*-1	0	1	3	5	6	6	7	8	8	1 : 16.59	1 : 10.52-1 : 26.16
sh*CD24*-2	0	1	2	4	6	6	7	8	8	1 : 18.57	1 : 11.78-1 : 29.27

J

HepG2 LCSC$^{CD24\ KD}$											
Frequency of tumor-sphere formation	Number of cell seeded each well									Stem cell frequence	
Dose	1	2	4	8	16	32	64	128	256	Estimate	Upper and lower limmits
sh*CD24*-NC	2	3	5	7	7	8	8	8	8	1 : 4.98	1 : 3.11-1 : 7.96
sh*CD24*-1	0	1	3	5	6	6	7	8	8	1 : 19.1	1 : 10.98-1 : 27.37
sh*CD24*-2	0	1	2	4	6	6	7	8	8	1:18.99	1 : 12.05-1 : 29.92

Supplementary Figure S4. SETD1B regulates LCSC stemness via CD24 and downstream N-cadherin.

(A) Single-cell RNA sequencing (GSE125449) analysis of *ADH1C*, *BCAT1*, *SHCBP1*, *CEACAM6*, *ABCB1*, *SLC28A2*, and *CEACAM5* genes expression across distinct

cellular clusters. (B) Spatial transcriptomics analysis (HRA000437) of *CD24* and *CEACAM6* expression in liver tumor-adjacent tissue and HCC tissue. (C) The pan-cancer-view of *CD24* and *CEACAM6*. (D) Overall survival prognosis of *CD24* and *CEACAM6* in LIHC. (E) Gene expression of *CD24* in HCCLM3 and HepG2 LCSCs after *SETD1B* KD. (F) (G) Detection and statistical analysis of the ability of HCCLM3 and HepG2 LCSCs to form clones after *CD24* KD. (H) Correlation of *CD24* with *N-cadherin* gene expression. (I) (J) Limited dilution analysis for detecting spheroid formation frequency in HCCLM3 LCSCs and HepG2 LCSC after *CD24* KD for 15 days. *$p < 0.05$, **$p < 0.01$, ***$p < 0.001$.

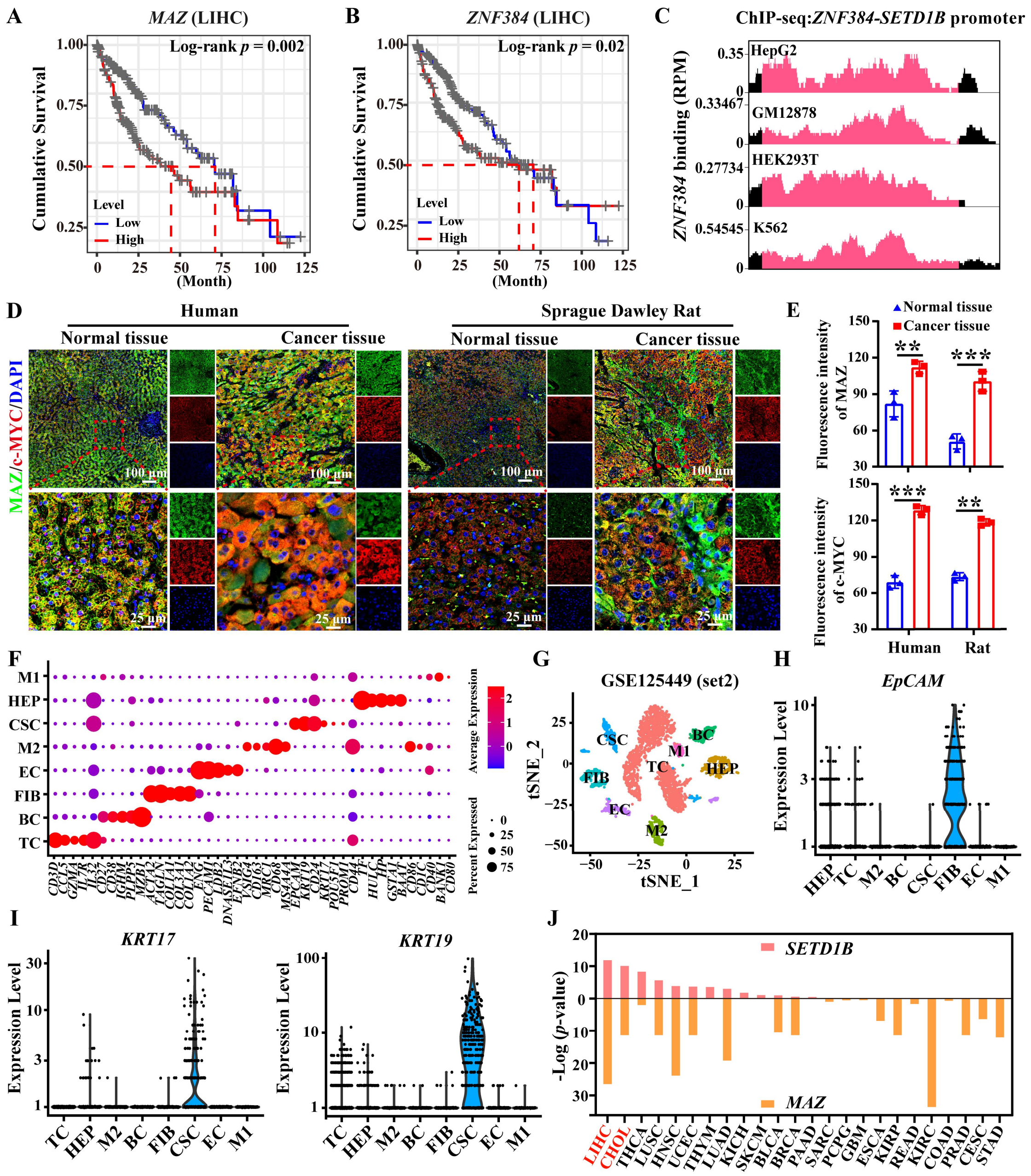

Supplementary Figure S5. MAZ expression is correlated with liver cancer stemness.

(A) Overall survival prognosis of *MAZ* in HCC. (B) Overall survival prognosis of *ZNF384* in HCC (C) *SETD1B* enrichment at *ZNF384* binding sites (reads per million). (D) IF staining of MAZ and c-MYC in normal and cancer tissues. (E) The statistical assessment of the mean fluorescence of MAZ and c-MYC in tumor tissues from each group. (F) Visualization of single-cell clusters (GSE125449) by R. (G) The tSNE plot

for the analysis of cellular subpopulations in HCC (GSE125449 set2). (H) (I) Expression of stemness-related markers (*EpCAM*, *KRT17* and *KRT19*) in single-cell data (GSE125449). (J) Differential expression (*p*-value) sorting of *SETD1B* and *MAZ* between normal and tumor tissues.

A B

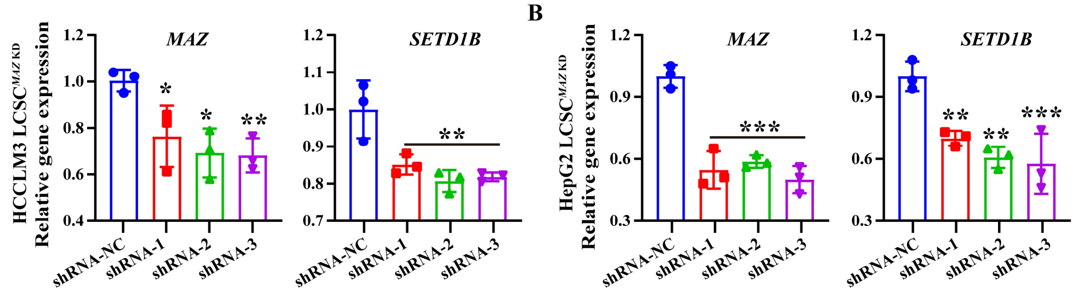

C

HCCLM3 LCSC $^{MAZ\ KD}$											
Frequency of tumor-sphere formation	Number of cell seeded each well									Stem cell frequence	
Dose	1	2	4	8	16	32	64	128	256	Estimate	Upper and lower limmits
sh*MAZ*-NC	4	5	5	6	7	8	8	8	8	1: 4.16	1:2.59 - 1:6.67
sh*MAZ* -1	4	4	4	4	5	7	7	8	8	1: 9.34	1:5.90 - 1:14.80
sh*MAZ* -2	2	2	3	4	5	7	7	8	8	1:12.05	1:7.62 - 1:19.04
sh*MAZ* -3	3	3	4	4	6	6	6	8	8	1:13.27	1:84 - 1:20.96

D

HepG2 LCSC $^{MAZ\ KD}$											
Frequency of tumor-sphere formation	Number of cell seeded each well									Stem cell frequence	
Dose	1	2	4	8	16	32	64	128	256	Estimate	Upper and lower limmits
sh*MAZ*-NC	4	5	5	6	7	8	8	8	8	1:3.22	1:1.99-1:5.21
sh*MAZ* -1	4	4	4	4	5	7	7	8	8	1:14.23	1:9.01-1:22.46
sh*MAZ* -2	2	2	3	4	5	7	7	8	8	1:16.86	1:10.69-1:26.59
sh*MAZ* -3	3	3	4	4	6	6	6	8	8	1:12.26	1:7.70-1:19.38

Supplementary Figure S6. *MAZ* depletion in LCSCs leads to reduced stem cell sphere-forming capabilities.

(A) (B) Genes expression of *MAZ* and *SETD1B* in HCCLM3 and HepG2 LCSC after *MAZ* KD. (C) (D) Limited dilution analysis for detecting spheroid formation frequency in HCCLM3 LCSCs and HepG2 LCSCs after *MAZ* KD for 15 days.

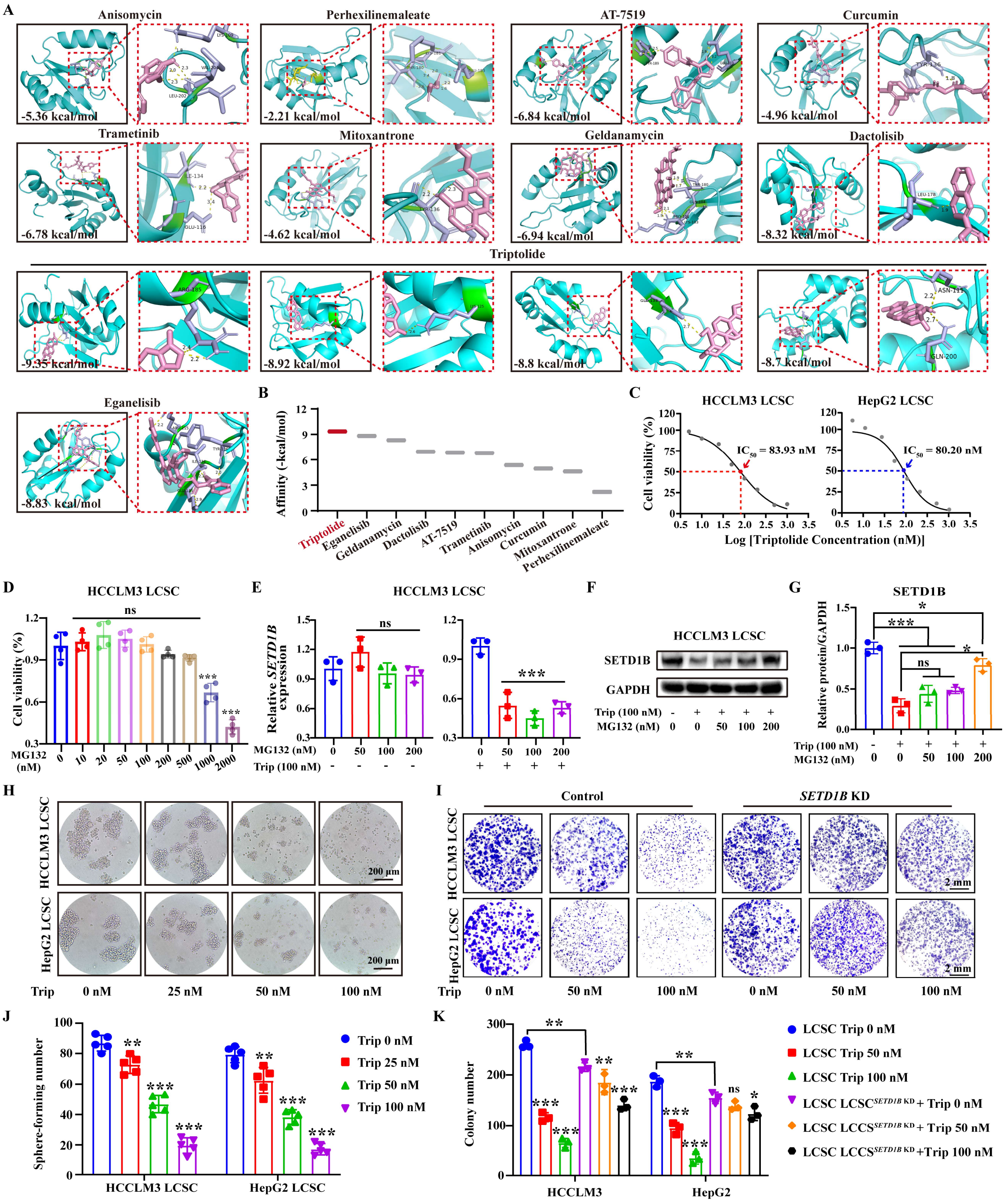

Supplementary Figure S7. Triptolide inhibits HCC progression by targeting SETD1B in LCSC.

(A) Molecular docking results of Triptolide and other drugs with SETD1B, respectively. (B) Quantitative assessment of SETD1B-drugs binding affinities by AutoDock. (C) Detection of IC_{50} for Trip treatment on HCCLM3 and HepG2 LCSCs. (D) The effect of MG132 on the activity of HCCLM3 LCSC. (E) Gene expression of *SETD1B* in HCCLM3 LCSC treated with or without MG132 (proteasome inhibitor) and Trip. (F) Protein expressions of SETD1B with or without Trip and MG132 (proteasome inhibitor)

in HCCLM3 LCSCs. (G) Quantitative image analysis of proteins expression for SETD1B. (H), (J) Detection and quantification of the ability of HCCLM3 LCSCs and HepG2 LCSCs to form spheroids after Trip treatment. (I), (K) Detection and quantification of the ability of HCCLM3 and HepG2 LCSCs with or without *SETD1B* KD to form clones after Trip treatment. * $p < 0.05$, ** $p < 0.01$, *** $p < 0.001$, ns: no significant difference.

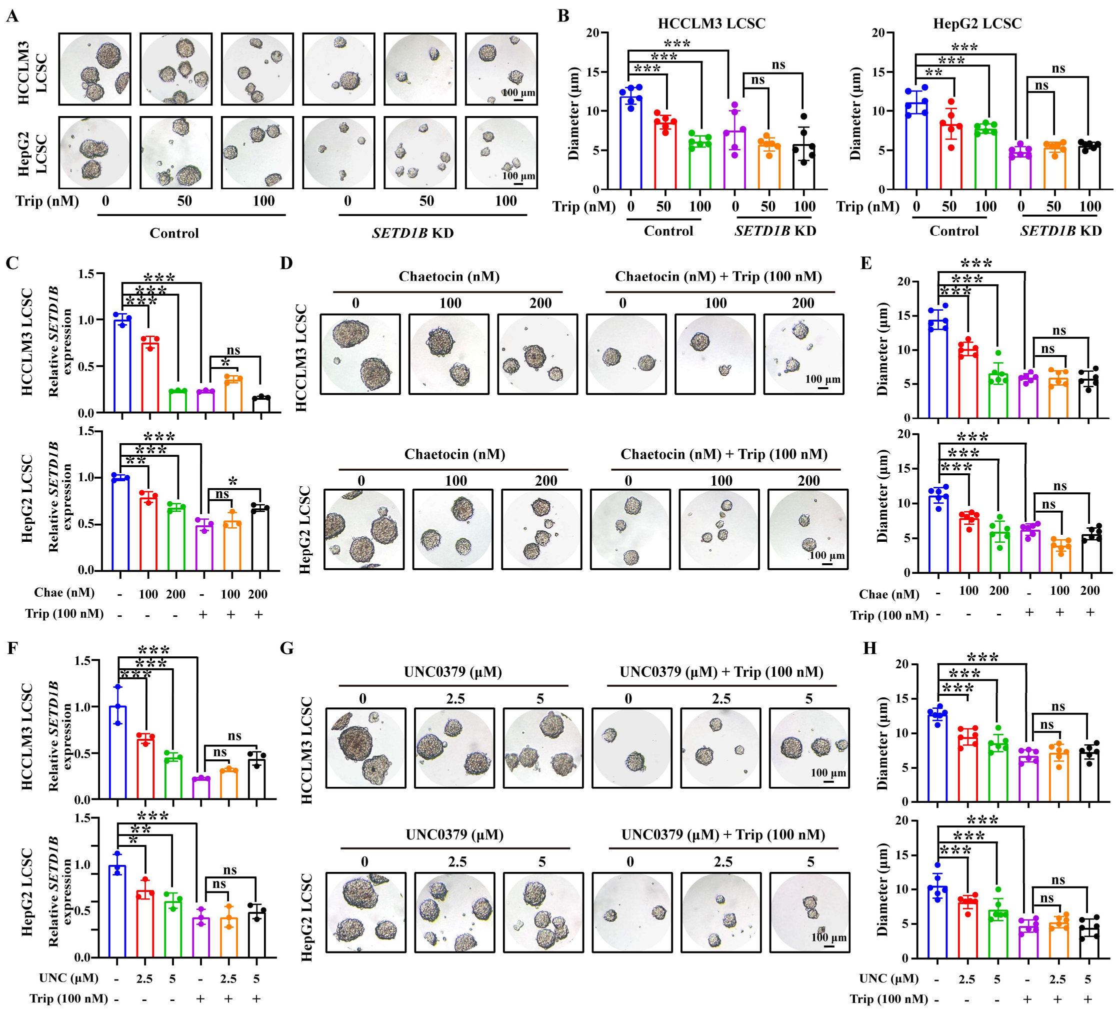

Supplementary Figure S8. Triptolide inhibits tumor sphere formation by targeting SETD1B in LCSCs.

(A) Spheroid formation ability of HCCLM3 and HepG2 LCSCs after Trip treatment. (B) Quantification of the diameters of HCCLM3 and HepG2 LCSCs spheroids after Trip treatment. (C) Gene expression of *SETD1B* in HCCLM3 and HepG2 LCSCs treated with chaetocin and Trip. (D) Spheroid formation ability of HCCLM3 and HepG2 LCSCs after chaetocin and Trip treatment. (E) Quantification of the diameters of HCCLM3 and HepG2 LCSCs after chaetocin and Trip treatment. (F) Gene expression of *SETD1B* in HCCLM3 and HepG2 LCSCs treated with UNC0379 and Trip. (G) Spheroid formation ability of HCCLM3 and HepG2 LCSCs after UNC0379

and Trip treatment. (H) Quantification of the diameters of HCCLM3 and HepG2 LCSCs spheroids after UNC0379 and Trip treatment. * $p < 0.05$, ** $p < 0.01$, *** $p < 0.001$, ns: no significant difference.

Tables

Table S1. List of antibodies and dilution factors for full text

Antibody	Cat No.	Company	Applications	Dilution
SETD1B	55005-1-AP	Proteintech	IF	1:100
SETD1B	Ab300748	Abcam	WB	1:1000
MAZ	101-10968	RayBiotech	IF/WB	1:100/1:1000
Nanog	14295-1-AP	Proteintech	WB	1:1500
OCT4	60242-1-Ig	Proteintech	IF/WB	1:200/1:5000
CD44	512410	Zenbio	WB	1:1000
CD90	555595	BD Biosciences	FC	1:50
CD133	18470-1-AP	Proteintech	WB	1:2000
CD133	567909	BD Biosciences	FC	1:50
c-MYC	67447-1-Ig	Proteintech	IF/WB	1:200/1:5000
EpCAM	21050-1-AP	Proteintech	IF/WB	1:100/1:2000
CD24	A25359	Abclonal	IF/WB	1:1000
N-cadherin	66219-1-Ig	Proteintech	WB	1:5000
BAX	342772	Zenbio	WB	1:1000
BCL2	15071	Cellsignal	WB	1:1000
Ki67	AF1738	Beyotime	IF	1:100
GAPDH	10494-1-AP	Proteintech	WB	1:20000

Table S2. List of gene primer sequences

Gene	Forward (5'->3')	Reverse (5'->3')
MAZ	CTGCCTTGGAGAAGAAGACA	ATAGCACCCGAGGGGACCCG
SOX2	AACCAGCGCATGGACAGTTA	GACTTGACCACCGAACCCAT
CD44	CAGCTCATACCAGCCATCCA	AGGTCCTGCTTTCCTTCGTG
CD133	TGACAAGCCCATCACAACATT	CGCCTGAGTCACTACGTTGC
Nanog	TACCTCAGCCTCCAGCAGAT	CTTCTGCGTCACACCATTGC
OCT4	GTGGAGAGCAACTCCGATGG	TGGTCGTTTGGCTGAATACCT
c-MYC	CCCTCCACTCGGAAGGACTA	GCTGGTGCATTTTCGGTTGT
EpCAM	AGCAGTTGTTGCTGGAATTGT	AGTTCCCTATGCATCTCACCC
CD24	GAACAAAGCAAGGGCTTCGG	AAATCTGCGTGGGTAGGAGC
SETD1B	Purchased from Beyotime	
GAPDH	AGGRGGAGGAGTGGGTGTCGCTGTT	CCGGGAAACTGTGGCGTGATGG

Table S3. List of shRNA sequences

Name	shRNA sequence	Vector Name
shRNA control	CCG**GGGTTCTCCGAACGTGTCACGT**CTC-GAG**ACGTGACACGTTCGGAGAACC**TTTT	pLKO.1-CMV-copGFP-PURO
shRNA targeting human *SETD1B*-1	CCGG**GCCGCCACGAACATCATTATG**CTC-GAG**CATAATGATGTTCGTGGCGGC**TTTT	pLKO.1-CMV-copGFP-PURO
shRNA targeting human *SETD1B*-2	CCGG**ACATGCGGGAGAAGCGTTATG**CTC-GAG**CATAACGCTTCTCCCGCATGT**TTTT	pLKO.1-CMV-copGFP-PURO
shRNA targeting human *SETD1B*-3	CCGG**TCGAGTACGTGGGCCAGAATA**CTC-GAG**TATTCTGGCCCACGTACTCGA**TTTT	pLKO.1-CMV-copGFP-PURO
shRNA targeting human *MAZ*-1	CCGG**GAGTTCAAGAACGGCTACAAT**CTC-GAG**ATTGTAGCCGTTCTTGAACTC**TTTT	pLKO.1-CMV-copGFP-PURO
shRNA targeting human *MAZ*-2	CCGG**GATGCTGAGCTCGGCTTATAT**CTC-GAG**ATATAAGCCGAGCTCAGCATC**TTTT	pLKO.1-CMV-copGFP-PURO
shRNA targeting human *MAZ*-3	CCGG**TCTGTGAGCTCTGCAACAAAG**CTC-GAG**CTTTGTTGCAGAGCTCACAGA**TTTT	pLKO.1-CMV-copGFP-PURO
shRNA targeting human *CD24*-1	CCGG**GCAGTCAACAGCCAGTCTCTT**CTC-GAG**AAGAGACTGGCTGTTGACTGC**TTTT	pLKO.1-CMV-copGFP-PURO
shRNA targeting human *CD24*-2	CCGG**CTTCTGCATCTCTACTCTTAA**CTC-GAG**TTAAGAGTAGAGATGCAGAAG**TTTT	pLKO.1-CMV-copGFP-PURO

Table S4. List of re-analyzed datasets and their sources

Data type	Number	Source
Bulk RNA sequencing	GSE141503	Gene Expression Omnibus (GEO) Database
ScRNA sequencing	GSE125449	Gene Expression Omnibus (GEO) Database
Spatial Transcriptomics	HRA000437	Genome Sequence Archive (GSA) for Human
